# The role of macrophage plasticity in neurodegenerative diseases

**DOI:** 10.1186/s40364-024-00624-7

**Published:** 2024-08-13

**Authors:** Hongyue Ma, Mingxia Zhu, Mengjie Chen, Xiuli Li, Xinhong Feng

**Affiliations:** Department of Neurology, Beijing Tsinghua Changgung Hospital, School of Clinical Medicine, Tsinghua University, Beijing, 102218 China

**Keywords:** Macrophages, Neurodegenerative disease, Immune cells, Gene expression, Imaging methodologies

## Abstract

Tissue-resident macrophages and recruited macrophages play pivotal roles in innate immunity and the maintenance of brain homeostasis. Investigating the involvement of these macrophage populations in eliciting pathological changes associated with neurodegenerative diseases has been a focal point of research. Dysregulated states of macrophages can compromise clearance mechanisms for pathological proteins such as amyloid-β (Aβ) in Alzheimer’s disease (AD) and TDP-43 in Amyotrophic lateral sclerosis (ALS). Additionally, recent evidence suggests that abnormalities in the peripheral clearance of pathological proteins are implicated in the pathogenesis and progression of neurodegenerative diseases. Furthermore, numerous genome-wide association studies have linked genetic risk factors, which alter the functionality of various immune cells, to the accumulation of pathological proteins. This review aims to unravel the intricacies of macrophage biology in both homeostatic conditions and neurodegenerative disorders. To this end, we initially provide an overview of the modifications in receptor and gene expression observed in diverse macrophage subsets throughout development. Subsequently, we outlined the roles of resident macrophages and recruited macrophages in neurodegenerative diseases and the progress of targeted therapy. Finally, we describe the latest advances in macrophage imaging methods and measurement of inflammation, which may provide information and related treatment strategies that hold promise for informing the design of future investigations and therapeutic interventions.

## Background

Macrophages were initially described in the scholarly works of Elie Metchnikoff, where they were characterized as cellular sentinels and facilitators of healing processes in response to infections and tissue injuries [1]. Wang et al. [2] discovered that the dynamic development of macrophages originating from embryonic stages across various tissues and organs, alongside the differentiation of distinct macrophage subtype populations, is orchestrated by subtype-specific transcription factors. These tissue-specific macrophages, commonly referred to as “resident macrophages,” encompass diverse cell types, such as microglia within the central nervous system (CNS), Kupffer cells in the liver, Langerhans cells in the epidermis, osteoclasts in bone tissue, and macrophages residing in the intestinal tract. Furthermore, certain macrophage subsets are distributed across multiple organs, as exemplified by perivascular macrophages (PVMs) situated on or in close proximity to the endothelial lining of blood vessels, where they execute a multitude of vital functions pertinent to vascular homeostasis [3–5]. Resident macrophages integrate signals derived from diverse environmental sensors to coordinate adaptive cellular responses crucial for the growth, remodeling, and maintenance of specialized tissue cell homeostasis [1, 6–11].

Macrophage efficiency decreases progressively with age. Functionally, factors secreted by senescent cells, termed Senescent Associated Secretory Phenotype (SASP), promote chronic inflammation and can induce the accumulation of M1-type macrophages in tissues, characterized by elevated concentrations of mediators such as IL-6, TNFα and C-Reactive Protein (CRP). Chronic inflammation ultimately leads to macrophage senescence characterized by a significant increase in SASP components (TNF-α, IL-6 and IL-1β), reduced levels of glycolysis and oxidative phosphorylation leading to energy depletion, defects in resistance to viral infections, and decreased phagocytosis. In molecular biology, the expression of mitochondrial calcium unidirectional transport protein (MCU) and its regulatory subunit MICU1, a core gene of the mitochondrial Ca^2+^ (mCa^2+^) signaling pathway, is inversely correlated with age. Correlative studies have shown that the mCa^2+^ uptake capacity of macrophages decreases significantly with age. Reduced mCa^2+^ uptake amplifies cytoplasmic Ca^2+^ oscillations and enhances downstream activation of the nuclear factor kappa B. Inflammation-driven tissue-resident macrophages polarize to an M1-like state [12, 13]. Furthermore, Seegren et al. [12] conducted a comprehensive analysis of 700 human blood transcriptomes, revealing a conspicuous trend toward age-related low-grade inflammation. Notably, monocytes emerge as pivotal mediators of chronic low-grade inflammation due to their capacity to differentiate into macrophages, thereby perpetuating low-grade inflammation and sustaining the inflammatory cascade response over extended durations. Moreover, specialized resident macrophages ubiquitously populate all tissues and organs, serving as central regulators of local inflammation and homeostasis. Prolonged exposure of the human body to the inflammatory milieu can significantly contribute to the onset and progression of age-associated pathologies, such as neurodegenerative and cardiovascular metabolic diseases. Hence, comprehending macrophage physiology and elucidating its role in neurodegenerative diseases is paramount for addressing these health challenges.

Macrophages, regarded as the paramount immune cells in the body, are omnipresent from their inception to the culmination of disease progression and exert a significant influence on the pathophysiology of CNS inflammation, thereby precipitating neurodegenerative diseases. Despite the protective function of the blood–brain barrier (BBB) in safeguarding the CNS against immune activation, its permeability escalates during episodes of inflammation, rendering the brain susceptible to infections. Enhancing our understanding of inflammatory mediators, notably cytokines, is imperative for elucidating these intricate mechanisms [14]. Hence, fostering the development of novel therapeutic approaches tailored to combat neurodegenerative diseases is imperative. Our objective is to provide a conceptual synthesis delineating the contemporary understanding of macrophage physiology and its pivotal role in neurodegenerative pathologies. Additionally, we endeavor to encapsulate the existing nexus between immunotherapeutic modalities and neurodegenerative disorders, thereby potentially guiding the blueprint for forthcoming investigations and the formulation of associated therapeutic interventions.

## The developmental process and polarization of macrophages

### Origin of macrophage development

Macrophages are among the most versatile and heterogeneous cell types and are ubiquitously distributed across nearly all mammalian tissues. They diligently surveil the local milieu, orchestrating intricate interactions to uphold homeostatic equilibrium [15–17]. The genesis and differentiation trajectory of resident macrophages from their nascent embryonic hematopoietic progenitors are poised to sculpt their functional attributes and contributions to both homeostatic regulation and pathological processes. Vertebrates exhibit at least three distinct types of hematopoietic progenitor cells characterized by disparate developmental timings and genetic origins. These lineages include primitive hematopoiesis, erythro-myeloid progenitors (EMPs), and hematopoietic stem cells (HSCs) [18].

Initially, the earliest wave of hematopoietic cell development encompasses primitive hematopoietic cells, which emerge in a manner independent of RUNX1 from the posterior plate mesoderm of the extraembryonic yolk sac. The absence of RUNX1 leads to a failure in the development of both mouse and human macrophages [11, 18, 19]. Subsequently, the second wave of development ensues with the emergence of EMPs, which originate from the yolk sac vascular endothelium. In mice, this process is contingent upon RUNX1 but operates independently of MYB and NOTCH1. Notably, EMPs can populate the fetal mouse liver. Studies [20–22] have shown that EMPs serve as precursors for various immune cells, including resident macrophages. These EMP-derived macrophage precursors (PreMacs) can colonize the entire embryo and subsequently differentiate into tissue-specific resident macrophages during organogenesis. PreMacs exhibit a fundamental transcriptional profile characterized by the expression of core transcription factors such as PU.1, cMAF, and interferon regulatory factor 8 (IRF8).

In contrast, HSCs represent the progenitor cells of the third developmental wave and are the subject of extensive study. Originating from mesoderm-derived aorta–gonad–mesonephros (AGMs), HSCs initially migrate to the fetal liver during embryonic development before eventually colonizing the bone marrow prior to birth. Unlike EMPs, HSCs possess self-renewal capabilities and have the capacity to differentiate into diverse erythroid, lymphoid, and myeloid cell lineages, including macrophages [11, 18].

While most studies indicate that HSCs do not significantly contribute to the proliferation of tissue-resident macrophages, tissue-resident macrophages typically exhibit a longer lifespan than macrophages derived from HSCs, which generally have a shorter lifespan [23–25]. Hence, EMPs have emerged as a primary reservoir for tissue-resident macrophages, contributing to a diverse array of tissue-specific macrophage populations. These populations include microglia in the CNS, Kupffer cells in the liver, Langerhans cells in the epidermis, osteoblasts in bone tissue, and intestinal macrophages, among others. Additionally, various subsets of macrophages are dispersed throughout multiple organs, as exemplified by PVMs, which localize along or in close proximity to blood vessel surfaces.

Tissue-resident macrophages establish intricate interactions with diverse cell types within their respective tissue microenvironments, playing specialized roles in antigen presentation. Disruptions of these functions can be implicated in organ-specific diseases. For instance, large peritoneal macrophages serve as resident defenders in the peritoneal cavity, shielding against microbial intrusion and inflammation while bolstering B cell activity. In contrast, small peritoneal macrophages, which originate from the bone marrow, exhibit a proinflammatory phenotype [26, 27]. Studies have shown that roughly half of the small peritoneal macrophages within the peritoneal cavity (PerC) express L-selectin CD62L. This finding lends support to the notion that, when appropriately stimulated, small peritoneal macrophages can migrate to lymph nodes and function as antigen-presenting cells [28]. Kupffer cells, as liver-resident macrophages, play a pivotal role in iron metabolism [29–31]. The differentiation of alveolar macrophages (AMs) requires granulocyte macrophage colony-stimulating factor (GM-CSF), which depends on the transforming growth factor beta receptor (TGF - β R) signaling pathway. Mechanistically, the TGF - β R signaling pathway leads to upregulation of PPAR - γ, which is a necessary characteristic transcription factor for the development of AMs [32, 33]. Macrophages residing in the kidneys can produce a strong myd88 dependent inflammatory response when encountering immune complexes containing immunostimulatory virus-like nucleic acids, resulting in recruitment of white blood cells and type III hypersensitivity reactions [34]. Osteoclasts secrete insulin-like growth factor 1 (IGF1), and upon bone resorption, they release transforming growth factor beta (TGFβ) and IGF1 into the bone matrix. These factors stimulate osteoblast activity and promote bone formation [18, 35–39]. Severe osteoclast destruction can disrupt bone remodeling, leading to conditions such as osteoporosis, increased bone density, and skeletal deformities. These changes may affect internal hematopoietic bone cavities and neuronal tissues, potentially causing neurological symptoms and hematological defects [18, 35, 37, 40, 41].

Consequently, comprehending the activation and mechanistic pathways of macrophages is critical for both preventing and treating such complications, thereby offering therapeutic avenues to improve the prognosis of future clinical patients.

### Gene expression in macrophages

Franklin Mall and other scientists have divided the first eight weeks of human embryonic development into 23 Carnegie stages, or Carnegie stage (CS1-CS23). During days 14–21 after fertilization (CS5-CS8), cells in this stage undergo germ layer specialization to form the early trichoblasts, contributing to the development of all organs and systems in our body. Between developmental stages CS13 and CS14, the head predominantly harbors immune cells of the early macrophage (Mf) subtype, comprising YSdMP_AFP^high^, YSdMP_AFP^low^, and S100B^+^ ACY3^+^ MP progenitors, and primitive head-enriched Mf progenitors (HeMPs). However, a minor proportion (3.8%) of microglia expressing markers such as P2RY12, TMEM119, SALL1, and C3 was also detected. In the liver, a substantial proportion of S100B^+^ ACY3^+^ MPs was observed from CS13 to CS18 and gradually declined thereafter, coinciding with the emergence of Kupffer cells from CS19 onward. Red marrow macrophages appear in the spleen at 10 postconceptional weeks (PCW). In the gut, from CS13 to CS20, Mfs expressing YSdMP_AFP^high^, YSdMP_AFP^low^, S100B^+^ ACY3^+^ MP, and prePraM constitute the majority. After 9 PCW, two intestinal Mf isoforms (CD209^+^ and CD207^+^) emerge, with CD209^+^ Mfs becoming predominant. From CS19 onward, Pre-PraM, PraM, and adrenal-specific Mf constitute the majority of the adrenal Mfs. In the female gonads, the majority of immune cells are either Pre-PraM or PraM cells [2, 5, 18].

**Fig. 1:**
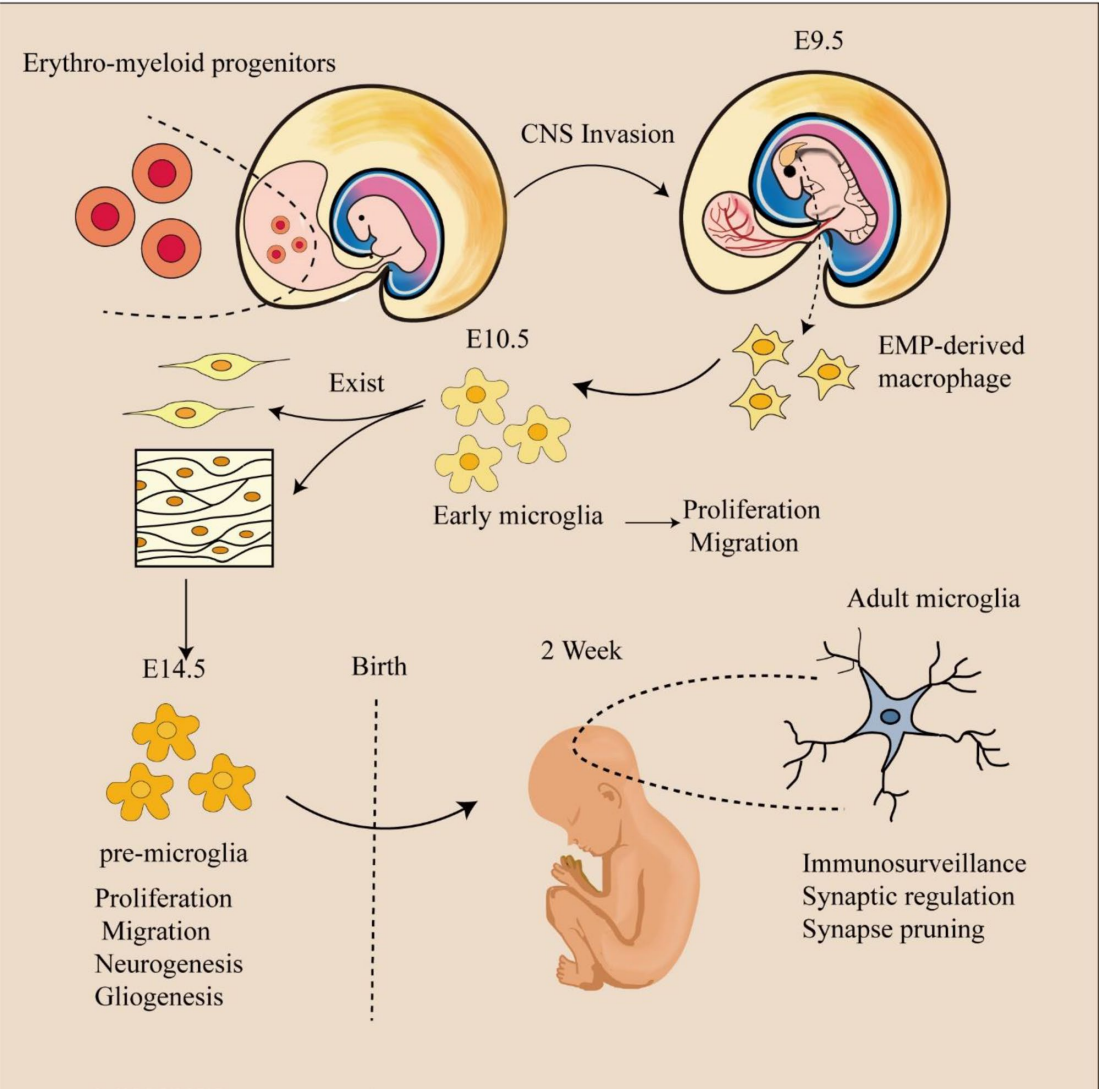
Microglial development. Microglia emerge from yolk sac precursors, and PreMacs are able to colonize the whole embryo and differentiate into tissue-specific resident macrophages during organ development. They colocate in the developing brain around E9.5. At approximately E14.5, a transition from early to premicroglia occurs, with premicroglia progressively acquiring adult properties after birth and different developmental phenotypes

Research [2] has shown that yolk sac-derived macrophage precursors (YSdMPs) can differentiate into primitive macrophages (PraMs), microglia, or microglia-like cells during development. Notably, the perivascular space has been identified as a niche where YSdMPs can differentiate into PraMs. Furthermore, PraMs have been found to play a regulatory role in angiogenesis within this perivascular environment(Fig. 1).

### Macrophage receptors

Macrophages serve as vigilant guardians of the local microenvironment, diligently maintaining homeostasis through the expression of an extensive array of sensing molecules. These receptors include scavenger receptors, pattern recognition receptors, nuclear hormone receptors, and cytokine receptors, which collectively equip macrophages with the ability to monitor tissue integrity and respond swiftly to infection or tissue damage. Moreover, the diverse functions of macrophages across various tissues are reflected in their distinct phenotypic profiles. For instance, alveolar macrophages, blast cells, microglia, and osteoblasts exhibit tissue-specific functions and phenotypes, suggesting that signals originating from the local tissue milieu may influence the development of tissue-specific macrophage phenotypes [5]. Upon activation by polysaccharides, macrophages initiate a cascade of intracellular signaling pathways by binding to cell-surface polysaccharide receptors. This activation leads to the release of inflammatory mediators such as TNF-α, IL-1β, and IL-6, thereby modulating the immune response. The key polysaccharide receptors involved in this process include Toll-like receptors (TLRs), the mannose receptor (MR), Dectin-1, scavenger receptors (SRs), complement receptor 3 (CR3), and other related receptors.

Toll-like receptor homologs, as type I transmembrane proteins, exhibit a tripartite structure comprising the extracellular region, the cytoplasmic region, and the transmembrane region. The transduction mechanisms of the TLR family predominantly involve two pathways: the myeloid differentiation factor 88 (MyD88)-dependent pathway and the MyD88-independent pathway. Notably, the MyD88-dependent pathway is shared among all TLR signaling pathways, except for TLR3. Upon encountering pathogen-associated molecular patterns (PAMPs), the extracellular segment of TLRs recognizes these ligands, prompting a conformational change in the Toll/interleukin-1 receptor (TIR) domain at the C-terminus. Subsequently, this conformational shift facilitates the recruitment of MyD88 or other adaptor proteins [42, 43].

Following PAMP recognition, MyD88 recruits interleukin-1 receptor-associated kinase (IRAK) via its N-terminal death domain (DD). This recruitment activates signaling molecules such as tumor necrosis factor receptor-associated factor 6 (TRAF6), β-transforming growth factor-activated factor, and β-transforming growth factor-activated protein kinase (TAK1). TAK1, along with its binding partners TAK1-binding proteins 1 and 2 (Tables 1 and 2), initiates downstream signaling cascades, leading to the activation of nuclear factor kappa-light-chain-enhancer of activated B cells (NF-κB) or activator protein-1 (AP-1). Consequently, this cascade induces the expression of inflammatory cytokines such as interleukin-1 (IL-1), IL-6, IL-8, IL-12, and tumor necrosis factor-alpha (TNF-α) [42].

The scavenger receptor superfamily encompasses a diverse range of ligands and functions that play crucial roles in maintaining tissue homeostasis and contributing to innate immune functions. SRs are categorized as pattern recognition receptors (PRRs) that are capable of recognizing both damage-associated molecular patterns (DAMPs) and PAMPs, as well as various structural properties, ligands, and functions. Currently, scavenger receptor A1 (SR-A1) and CD36 are the primary mediators, accounting for a significant proportion (75–90%) of the phagocytosis and degradation of oxidized low-density lipoprotein (OX-LDL) by macrophages [44, 45]. Thus, understanding scavenger receptor action is potentially valuable for ameliorating the progression of atherosclerosis.

The MR, a C-type lectin predominantly expressed on macrophages and dendritic cells, plays a pivotal role in recognizing polysaccharide components within the cell wall. Upon interaction with its ligands, MR initiates a cascade of intracellular signaling events, leading to the activation of the transcriptional machinery. This activation, in turn, modulates the expression of various molecules, including nitric oxide (NO) and a spectrum of cytokines, such as interleukin-1 (IL-1), IL-6, granulocyte-macrophage colony-stimulating factor (GM-CSF), TNF-α, IL-12, IL-10, IL-1 receptor antagonist (IL-1ra), and IL-2 receptor (IL-RII). Thus, MR serves as a critical mediator of immune responses, contributing to the regulation of inflammatory and immunoregulatory pathways [46]. IL-4, IL-13, and IL-10 have been shown to enhance the expression of MR on peritonitis-recruited macrophages. Additionally, prostaglandin E (PGE), dexamethasone, and 1,25-dihydroxyvitamin D3 have been identified as agents capable of upregulating MR levels [47]. This upregulation leads to the effective activation of MR receptor‒ligand binding, resulting in increased macrophage phagocytosis and antigen presentation. These findings suggest promising therapeutic avenues for the treatment of inflammatory diseases.

The Dectin-1 receptor, alternatively referred to as the C-type lectin structural domain family 7 member A (CLEC7A) receptor, employs intricate mechanisms for the recognition of pathogenic bacteria. Among these mechanisms, the classical pathway involves Dectin-1 triggering of the Syk/Card9/NF-κB signaling cascade upon binding to the carbohydrate fiber β-glucan. This signaling pathway plays a pivotal role in the immune response against pathogens recognized by Dectin-1 [48]. The Dectin-1 receptor exhibits specificity in recognizing β(1–3) and β(1–6) linkages found in β-glucan molecules, with some studies suggesting potential binding to β(1–4) linkages as well [49].

Moreover, Dectin-1 can distinguish between granular and soluble beta-glucans. This distinguishing ability enables the receptor to modulate the inflammatory response, whereby Dectin-1 bound to granular β-glucans localized in the vicinity of the pathogenic fungus can trigger an inflammatory response, while soluble β-glucans from distant cells infected with the fungus may not elicit the same response [50]. This differential response underscores the sophisticated regulatory role of Dectin-1 in coordinating immune responses against fungal pathogens. When particulate β-glucan binds to Dectin-1 receptors, it triggers the aggregation of Dectin-1 receptor populations, leading to reduced expression of regulatory receptors such as CD45 and CD148. In contrast, soluble β-glucan lacks the ability to aggregate Dectin-1 receptors and consequently exhibits a weaker capacity to activate Dectin-1 receptors. Remarkably, soluble β-glucan can even hinder the activation of Dectin-1 induced by granular β-glucan by interfering with Dectin-1 receptor binding and aggregation processes. This intricate interplay highlights the nuanced regulatory mechanisms governing Dectin-1-mediated immune responses and underscores the importance of the β-glucan particulate structure in modulating receptor activation [50]. Soluble dextran stimulates the production of interleukin-10 (IL-10), TNF-α, and interleukin-23 (IL-23) in dendritic cells. Additionally, soluble dextran enhances the production of interleukin-4 (IL-4) and IL-23 in dendritic cells stimulated with granular dextran. IL-4 is known to be associated with Th2 immune responses, which are traditionally considered detrimental to fungal clearance. This result suggests that the presence of soluble dextran may modulate dendritic cell responses toward a Th2 phenotype, potentially impacting the immune defense against fungal pathogens [51]. Hence, conducting in-depth studies on the dectin-1 receptor has significant research value, particularly concerning the treatment of fungus-related diseases. Understanding the intricate mechanisms by which Dectin-1 recognizes and responds to fungal pathogens can offer valuable insights into the development of novel therapeutic strategies. By elucidating the signaling pathways and immune responses triggered by Dectin-1 activation, researchers can explore innovative approaches for targeting fungal infections more effectively. Moreover, uncovering the regulatory mechanisms governing Dectin-1 function may pave the way for the development of targeted therapies that harness the immune system’s innate defenses against fungal pathogens. Therefore, continued investigation into the role of Dectin-1 in fungal immunity holds great promise for advancing our understanding and management of fungus-related diseases.

CR3 is a heterodimeric glycoprotein composed of two peptide chains, α and β, connected by a noncovalent bond, with molecular weights of 165 kDa and 95 kDa, respectively. CR3, a member of the integrin family of adhesion molecules, exhibits structural similarity to lymphocyte function-associated antigen-1 (LFA-1) and CR4. These molecules share identical β chains (designated CD18), while their α chains vary: CD11a for LFA-1, CD11b for CR3, and CD11c for CR4. Consequently, CR3 is commonly referred to as the CD11b/CD18 molecule [52].

**Fig. 2:**
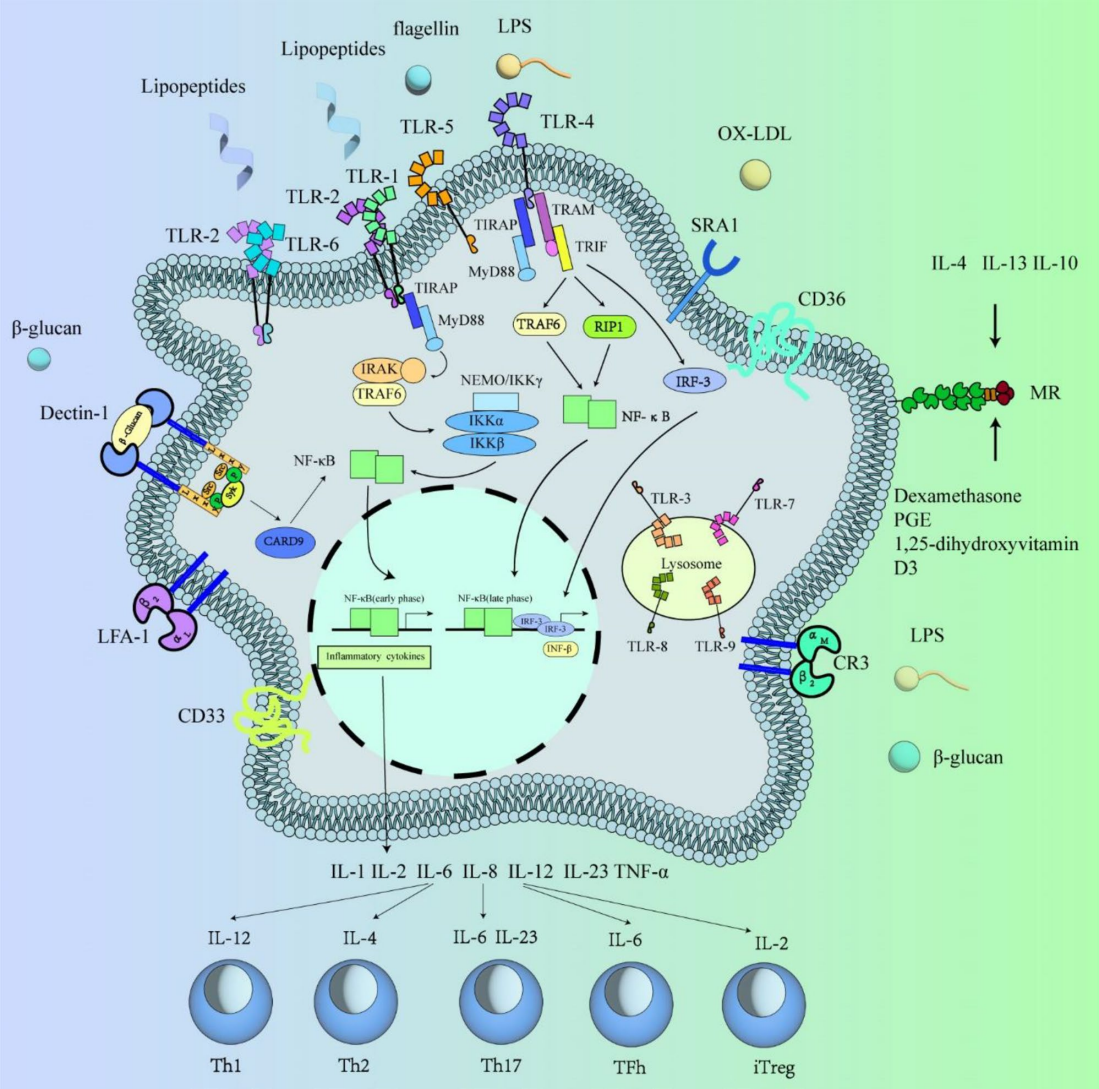
Macrophage receptors and their secreted factors. Polysaccharide-activated macrophages first bind to cell surface polysaccharide receptors, activate intracellular signaling pathways, mediate the release of inflammatory factors such as TNF-α, IL-1β, and IL-6, and subsequently enhance their immunomodulatory ability. The main polysaccharide receptors identified include TLRs, MR, Dectin-1, SR, and CR3. Inflammatory factors can activate different T-cell subsets

CR3 also has sites that bind to bacterial LPS and β-glucan in yeast cell walls. In inflammatory responses, CR3 mediates neutrophil adhesion to endothelial cells, facilitating their recruitment to sites of inflammation. Additionally, CR3 plays a crucial role in enhancing phagocytosis by promoting close contact between effector and target cells. This function is vital for effective anti-infection immunity, underscoring the significance of CR3 in host defense mechanisms against microbial pathogens [52, 53]. CD33 is prominently expressed on the surface of myeloid progenitor cells, mature monocytes, and macrophages. As a lectin, CD33 functions as a cell binding protein. Notably, it contains putative immunoreceptor tyrosine-based inhibitory motifs (ITIMs), which typically confer inhibitory properties to the receptor, regulating cellular activity. This inhibitory role of CD33 is significant in modulating the functions of myeloid cells, including monocytes and macrophages, thereby influencing immune responses and inflammatory processes [54](Fig. 2).

### Macrophage polarization states

Macrophages have high plasticity. They undergo morphological and functional changes under the influence of different tissue environments in the body. After tissue damage occurs, it causes inflammation, proliferation of locally recruited or resident macrophage populations, as well as significant phenotypic and functional changes occur under the regulation of growth factors and cytokines released in the local tissue microenvironment, inducing macrophage polarization [55]. IFN-γ or bacterial lipopolysaccharides (LPS) released by NK, Th1, etc. can activate macrophages to form M1 macrophages, which eliminate intracellular pathogens through type I inflammatory response, delayed hypersensitivity response, and cellular phagocytosis, while activating the body’s specific immune response; The IL-4 and IL-13 released by Th2 cells can activate macrophages to form M2 macrophages, which can synthesize and release a large amount of anti-inflammatory cytokines and immunosuppressive factors. Moreover, M2 macrophages have an inhibitory effect on the inflammatory response. M2 can be further subdivided into M2a, M2b, M2c, and M2d [56–59].

Macrophages differentiate into two distinct and functionally contrasting subtypes: classically activated (M1-type) macrophages, which are stimulated by LPS and interferon-gamma (IFN-γ), and alternatively activated (M2-type) macrophages, which are induced by IL-4 and IL-13. M1 macrophages exhibit robust secretion of proinflammatory factors such as TNF-α, IL-6, IL-12, chemokine ligand 2, MMP-2, and MMP-9, among others, thereby promoting Th1 cell polarization and recruitment, thus augmenting or sustaining inflammatory responses [60, 61]. Furthermore, chemokines prompt M1 macrophages to recruit inducible nitric oxide synthase (iNOS), which facilitates the production of nitric oxide (NO). Subsequently, NO activates the enzyme NADPH oxidase, leading to the release of reactive oxygen species (ROS) and other stimulatory factors. These compounds not only amplify the lesion but also activate additional immune cells to eliminate the pathogen [62]. In gene sequencing studies of macrophages induced in vitro to exhibit both M1 and M2 phenotypes, a notable finding was that the majority of upregulated genes in M1 macrophages were associated with endocytosis. This observation may help researchers elucidate the prevalence of M1 macrophages over M2 macrophages in the vascular microenvironment.

M2 macrophages are activated via IL-4 and IL-13, which trigger the activation of STAT6 through IL-4 receptor α (IL-4Rα). Subsequently, STAT6 activation stimulates Th2 cells to release cytokines such as IL-4, IL-5, and IL-13, which possess anti-inflammatory properties. Moreover, in addition to IL-4, IL-5, and IL-13, cytokines such as IL-10 can modulate M2 polarization by activating STAT3 through the IL-10 receptor (IL-10R) [57, 63].

Furthermore, M2 macrophages secrete mannose receptors, which play a pivotal role in attenuating inflammation and fostering tissue repair or remodeling processes [63, 64]. In situations where tissues experience persistent stimulation with inflammatory factors, the normal wound healing response becomes disrupted and the gradual accumulation of extracellular matrix ensues, culminating in the formation of scars or fibrosis. The progression of this pathological condition is a significant contributor to organ failure and mortality. Remarkably, the ability of macrophages to undergo M2 polarization serves as an effective countermeasure, mitigating the escalation of such detrimental states and contributing to the preservation of tissue homeostasis [63]. M2 macrophages can dedifferentiate into four distinct subtypes: 2a, 2b, 2c, and 2d. While M2b macrophages deviate from this pattern, the other three subtypes are known for their capacity to secrete specific chemokines and anti-inflammatory factors. These activities are associated with promoting the regression of inflammation and fostering tissue regeneration [65]. The M2a macrophage subpopulation can be induced by IL-4 and IL-13, leading to the production of elevated levels of CD206, decoy receptor IL-1 receptor II (IL-RII), and anti-IL-1 receptor (IL1Ra). Conversely, M2b macrophages can be activated by immune complexes (ICs), TLRs, and IL-1 receptor ligands, resulting in the release of cytokines such as IL-10, IL-1β, IL-6, and TNF-α [57].

**Fig. 3:**
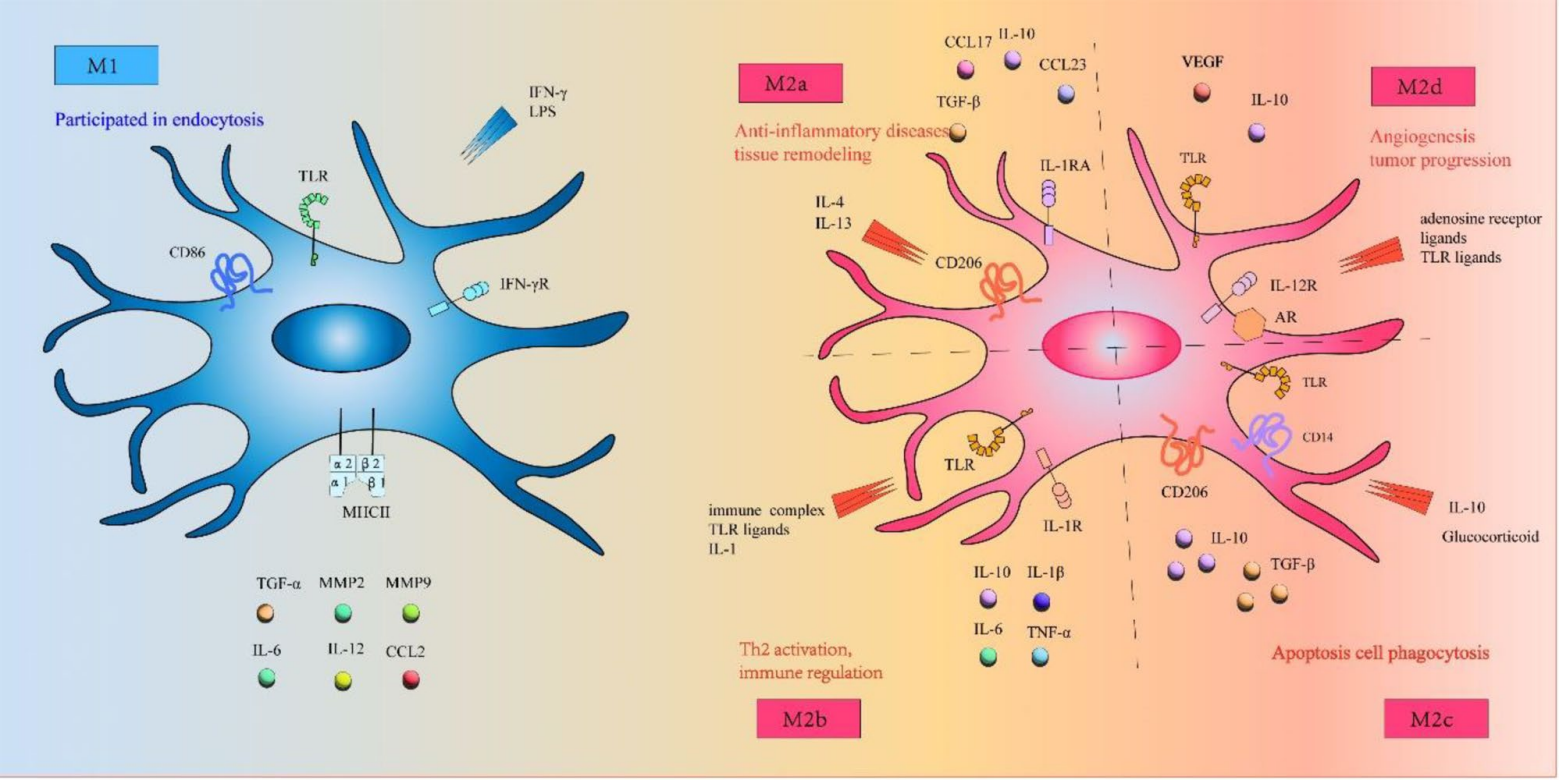
The structures, surface markers, cytokine secretion and biological functions of the M1 and M2 macrophage subpopulations are summarized. Macrophages differentiate into two heterogeneous and opposing macrophage subtypes: classically activated (“M1-type”) macrophages stimulated by LPS and IFN-γ and alternatively activated (“M2-type”) macrophages stimulated by IL-4 and IL-13. M2 macrophages can dedifferentiate into four subtypes: 2a, 2b, 2c and 2d. With the exception of M2b macrophages, the other three cell types are activated by the secretion of the appropriate chemokines and anti-inflammatory factors

On the other hand, M2c macrophages are induced by glucocorticoids and IL-10 and exhibit potent anti-inflammatory activity against apoptotic cells through the abundant secretion of IL-10 and TGF-β. Moreover, M2d macrophages are induced by Toll-like receptor (TLR) agonists or adenosine receptors, with adenosine receptor activation responsible for inhibiting the production of proinflammatory cytokines and inducing the release of anti-inflammatory cytokines (high IL-10, low IL-12) and vascular endothelial growth factor (VEGF). This characteristic confers proangiogenic properties consistent with those of tumor-associated macrophages [66]. Moreover, Hofbauer cells express CD209 (a marker of M2a macrophages), CD86 (a marker of M2b macrophages), HLA-DR (a marker of both M2a and M2b macrophages), CD206 (a marker of M2a and M2c macrophages), and CD14 (a marker of M2c macrophages) [67](Fig. 3).

### Macrophage typing detection technology and imaging technology

#### Macrophage typing detection technology

At present, macrophages can be isolated and analyzed from whole blood (PBMC isolation, macrophage analysis, monocyte differentiation into macrophages) or tissues. There are various methods for isolating macrophages, including magnetic bead cell sorting, density gradient separation, laser capture microanatomy, and flow cytometry sorting (FACS) [68]. After separation, macrophage function and phenotype can be analyzed and characterized through gene expression analysis, functional studies, evaluation of cytokine and chemokine generation, as well as protein expression and cell surface markers using immunofluorescence staining, immunoassay, flow cytometry, immunoblotting or polymerase chain reaction. Nevertheless, not every analytical method requires the isolation of macrophages [68]. In addition, when segmenting cell subtypes in single-cell data, based on different subtypes of M1 or M2 markers and summarizing cytokines together, a gene set of relevant subtypes can be obtained for scoring to distinguish macrophage subtypes [69].

Additionally, isolated macrophages can be further cultured and stimulated. In most cases, M1 macrophages can be reprogrammed into M2 macrophages through stimulation, and vice versa. The cultivation of macrophages can be used to stimulate or generate cell lysates or cell culture supernatants, which can be used for qPCR detection, immunoblotting, or various immunoassays (IA), including ELISA, magnetic bead based immunoassays, and PCR based immunoassays. Macrophage function can also be studied by detecting phagocytosis.

#### Imaging techniques for microglia/macrophages in vivo

Recent advancements in in vivo imaging techniques offer remarkable opportunities to noninvasively monitor the dynamic transformation of microglia/macrophages and track their interactions with surrounding cells or structures. These imaging modalities enable direct observations of microglial/macrophage involvement in CNS repair processes and facilitate the correlation of morphological changes with cellular behavior in the intact environment [70–72].

#### Two-photon excitation fluorescence microscopy (TPM)

Among these techniques, two-photon excitation fluorescence microscopy (TPM) stands out as a powerful tool for three-dimensional (3D) imaging of turbid tissues, allowing the visualization of cellular and subcellular structures and functions deep within biological samples [73]. While TPM has traditionally been instrumental in studying the roles of microglia/macrophages in specific repair processes, recent innovations have introduced a novel gradient light-field two-photon microimaging technique.

This cutting-edge approach requires only two-dimensional (2D) scans to acquire 3D information about the sample, significantly reducing laser-induced damage, it accurately reflects the true 3D fluorescence intensity of the sample and provides depth information with high precision. Gradient light-field two-photon microscopy exhibits exceptional suitability for three-dimensional imaging of living cells. In experiments observing the phagocytosis of fluorescent spheres by macrophages, rapid capture of the three-dimensional trajectories of fluorescent spheres both inside and outside macrophages has been achieved. Moreover, it facilitates precise quantification of the speed at which macrophages transport spheres, thereby offering valuable insights into cellular dynamics and interactions [74].

#### Magnetic resonance imaging (MRI) and single photon emission tomography (SPECT/CT)

Superparamagnetic iron oxide (SPIO) nanoparticles offer a valuable means for tracking various cell types via magnetic resonance imaging (MRI) within living organisms. In a previous study, macrophages were specifically labeled with SPIO nanoparticles and 111Indium (111In) for enhanced tracking. Inductively coupled plasma‒mass spectrometry (ICP-MS) was employed to quantify the iron content within macrophages. This approach enabled the monitoring of macrophage recruitment and disease activity in vivo, providing insights into their dynamics and involvement in pathological processes [75].

#### Positron emission tomography (PET)

PET imaging, a molecular imaging technique utilizing nuclide labeling and tracing, allows dynamic and continuous monitoring of various aspects, such as migratory localization, cell targeting, population expansion, cell activation, and immune functioning, of hyperactive immune cells in vivo. Despite its relatively lower imaging resolution (1–3 mm), PET offers exceptional imaging sensitivity. Radioligands that specifically bind to the translocator protein 18 kDa (TSPO) can be employed, with the isoquinoline carboxamide derivative [11 C]PK-11,195 being the most commonly used. [11 C]PK-11,195 is significantly distributed in activated microglia/macrophages across various models of cerebral ischemia and traumatic brain injury [70].

## Physiological function and immune regulation of resident macrophages in organisms

Tissue-resident macrophages constitute a conserved lineage in postnatal organisms and play pivotal roles in development, tissue maintenance, and overall organismal homeostasis through paracrine signaling. Across the trajectory from organogenesis to maturity, these macrophages establish enduring associations with specialized cell populations distinct from circulating monocytes and their derivatives. Given the diverse and sometimes contradictory functions exhibited by various macrophage subtypes, forthcoming investigations must discern the nuanced contributions of individual subpopulations to specific phenotypic outcomes. This conceptual framework not only facilitates the exploration of complex tissue physiology but also holds promise for unraveling the pathophysiological mechanisms and genetic underpinnings of multifaceted diseases such as dementia, obesity, autoimmune disorders, and cancer, thus paving the way for the discovery of novel therapeutic interventions [76].

### The physiology of microglia

Microglia, which serve as “macrophages” within the CNS. Widely dispersed throughout the CNS parenchyma, they constitute approximately 10% of the total glial cell population. As the primary immune component of the brain, microglia play indispensable roles not only in CNS development and maintenance but also in the regulation of pathological conditions, including neurodegenerative diseases, autoimmune disorders, and neurodevelopmental anomalies. Originating from EMPs within the embryonic yolk sac, microglia differentiate from PreMacs, which populate the entire embryo and undergo tissue-specific specialization during organogenesis [18, 77].

Microglia generally exist in two distinct states: resting and activated. In their resting state, microglia actively surveil the microenvironment and contribute to synaptic pruning, neurogenesis, and the regulation of neural networks without inducing an inflammatory response. During homeostasis, microglia typically display a “branching” morphology characterized by quiescent cell bodies and elongated, highly dynamic branches that maintain transient contact with peripheral neurons, blood vessels, and astrocytes. These branches allow them to continually “monitor” their surroundings and respond to any aberrant neuronal activity. These functions collectively contribute to the neuroprotective effects attributed to microglia [78]. Upon stimulation, microglia undergo rapid morphological changes, transitioning into either a “hypertrophic” or an “amoeboid” state. Transcriptomic analyses revealed that “hypertrophic” microglia, in response to inflammatory stimuli, express genes associated with neuronal maturation, synaptic transmission, and antigen presentation. On the other hand, “amoeboid” microglia express genes related to cell cycle progression, migration, and phagocytosis [57].

Microglial activation and subsequent inflammatory responses are initiated by various damage sensors, including TLRs, cytoplasmic DNA/RNA sensors, triggering receptor expressed on myeloid cells 2 (TREM2), receptor for advanced glycation end products (RAGE), P2 purinergic receptors, and complement and scavenger receptors. Activation involves alterations in cellular protrusions, increases in cell body size and proliferation, and enhanced phagocytosis, collectively referred to as microglial proliferation. This process is mediated through signaling pathways such as nuclear factor-kappa B (NF-κB), interferon regulatory factor 3 (IRF3), and mammalian target of rapamycin (mTOR) [79].

Depending on the extent and duration of injury, microglia differentiate into proinflammatory (M1) or anti-inflammatory (M2) phenotypes. Proinflammatory microglia secrete cytokines such as TNF, IL-1β, IL-6, interferon-β, and chemokines; upregulate damage sensors; and produce ROS and nitric oxide (NO). In contrast, anti-inflammatory microglia (M2) inhibit inflammation, downregulate certain damage sensors, promote tissue regeneration via the cytokines IL-4, IL-13, and IL-10, and phagocytose cellular debris and aggregated proteins via the upregulation of the scavenger receptors YM1 and CD206. Moreover, microglia can display various morphologies, including “senescent” and “rod-like” morphologies [80]. Senescent microglia exhibit distinctive features, including “bead-like” spherical swelling of their protrusions, the accumulation of lipofuscin (a marker of incomplete lysosomal degradation and endolysosomal stress), and dilation of the endoplasmic reticulum. As senescence progresses, microglial protrusions display uneven levels of IBA1 until the spherical cell body becomes completely detached from the cytoplasm. This detachment results in long, thin protrusions that were once part of the cytoplasm [57].

Previous studies have shown that the increased density of microglia with aging exacerbates neurodegenerative diseases, suggesting that aging microglia may become less responsive to chronic inflammatory stimuli and may lose their neuroprotective and phagocytic abilities [81, 82]. Slowing microglial senescence represents a novel diagnostic and therapeutic approach for treating neurodegenerative diseases [83].

#### Microglia in the central nervous system

A portion of early EMPs differentiate into microglial precursor cells in the yolk sac on the 9th day of embryo (E9), and microglial precursor cells are implanted into the embryonic brain starting from E9.5. The precursor microglia in the yolk sac can be distinguished based on their expression of PU.1 (also known as SPI1), CSF1R, CD45, high-level F4/80 (also known as ADGRE1), and CX3C chemokine receptor 1 (CX3CR1). The late stage EMPs produced in the yolk sac since E8 are implanted in the fetal liver, where they expand and produce myeloid (e.g. monocytes) and red blood cell lines [84–86]. By E10.5, microglia are observed within both cephalic lateral mesenchymal cells and neuroepithelial cells, albeit at a reduced density within the latter [87]. Embryonic microglial differentiation hinges upon the coordinated action of two pivotal myeloid transcription factors, namely, the transcription factor Pu.1 and IRF8. Following their ingress into the neural ectoderm, the embryonic microglial population undergoes a phase of proliferation and subsequent expansion throughout the developmental stages. The proliferation and differentiation processes of developing microglia critically rely on the expression of colony-stimulating factor 1 receptor (CSF1R). Notably, with the establishment of the blood–brain barrier at approximately E13.5, microglia within the brain are shielded from subsequent hematopoiesis originating from the fetal liver and bone marrow. Consequently, under homeostatic conditions, monocyte-derived cells do not supplant yolk sac-derived microglia. The transition from early microglia to premicroglia occurs at approximately E14.5, followed by a gradual acquisition of adult-like properties postnatally, with full maturation to adult microglia typically commencing around the second or third week of life [88]. The origin and migratory trajectories of early microglia play pivotal roles in shaping microglial populations and profoundly influence neurodevelopment and the maintenance of nervous system homeostasis. Understanding these processes holds significant promise for leveraging microglia as a noninvasive cellular therapy for gene delivery to the nervous system [89].

### Regulation of peritoneal macrophages

GATA6 has been implicated in the regulation of peritoneal macrophage-specific gene expression. Ghosn et al. [28] documented the presence of two distinct subpopulations within mouse peritoneal macrophages. Leptomeningeal macrophages (LPMs) and small peritoneal macrophages (SPMs) are two distinct subpopulations of peritoneal macrophages. LPMs are predominant and exhibit elevated F4/80 expression but low MHC class II (MHC-II) expression. In contrast, SPMs display lower levels of F4/80 but higher expression of MHC-II. Interestingly, during inflammatory responses, SPMs exhibit heightened expression of the chemokine receptor CCR2 mRNA [90]. The number of small peritoneal macrophages (SPM) in the peritoneal exudate of CCR2 knockout (KO) mice was notably decreased, while the number of LPMs was unaffected. This observation suggested that the majority of SPMs originate from the inflammatory monocyte population [26]. The distribution and function of macrophages within tissues are intricately regulated by various signals, including the vitamin A metabolite retinoic acid. Retinoic acid governs macrophage differentiation and function by activating the retinoic acid receptor (RAR). Moreover, signals originating from other tissue sources, such as peritoneal adipose tissue, also play a crucial role in regulating macrophage distribution and function within specific tissue compartments, such as the peritoneal cavity. These insights offer valuable perspectives for understanding the multifaceted roles and regulatory mechanisms of macrophages in tissue environments.

### Activation of meningeal macrophages

The meninges serve as a protective barrier surrounding the CNS. Within the complex layers constituting the meninges, the dura mater is notable for its abundance of tissue macrophages. These macrophages, akin to microglia, originate from yolk sac precursors and maintain vigilant surveillance of their surrounding environment [15, 91, 92]. Rua et al. [93] reported that upon infection of the meninges with lymphocytic choroid plexus meningitis virus (LCMV), antiviral cytokines produced within this tissue prompted the activation of meningeal macrophages. During the zenith of the infection, approximately one-third of CD206^+^ meningeal macrophages (MMs) displayed viral antigens and succumbed to cytotoxic CD8^+^ lymphocytes. The IFN-γ released by infected MMs promoted heightened MHC-II expression, facilitating CD4^+^ lymphocyte binding. By the 30th day postinfection, most LCMV-infected MMs were eradicated. Chemokine-recruited peripheral blood mononuclear cells replenished the MM niche across the meninges. Thus, in addition to the previously described epigenetic mechanisms regulating the activity of CNS-resident macrophages, infection can induce enduring alterations in CNS immunity by triggering the replacement of MMs with peripheral blood monocytes [15, 93–95]. Indeed, the replacement of tissue-resident dural macrophages with inflammatory monocytes has the potential to significantly impact CNS immunity, homeostasis, and neurological function.

### Immune regulation of macrophages in the spleen and liver

Macrophages play a crucial role in the clearance of apoptotic cells and in orchestrating a noninflammatory response [96, 97]. The development of the “waste disposal hypothesis” of autoimmunity, which is related to complement deficiency, highlights the pivotal role of tingible body macrophages, particularly those present in the spleen.

Macrophages play a central role in iron homeostasis, primarily in the spleen (red pulp) and liver (Kupffer cells), where they recycle iron obtained from hemoglobin. Damaged or senescent erythrocytes are degraded through macrophage phagocytosis and protein hydrolysis. In mice, erythromedullary macrophages exhibit the F4/80^+^ CD206^+^ CD11b^lo/-^ phenotype and selectively express the transcription factor Spi-C. The absence of Spi-C results in deficient splenic erythromedullary macrophages, impairing erythrocyte clearance and leading to iron accumulation in the red pulp [29]. Macrophages release hemoglobin and eventually iron, which can be stored as a ferritin complex or exported via iron transport proteins [98]. When hemoglobin is released into the circulation, such as upon damage to erythrocytes, it binds to haptoglobin. This complex is then recognized by the scavenger receptor CD163, facilitating its scavenging by macrophages [99]. A secondary system for the clearance of hemoglobin has been described, wherein hemoglobin binds to haptoglobin and is subsequently recognized by CD91 expressed on macrophages, facilitating its clearance [99]. The splenic marginal zone is characterized by an abundance of B cells and specialized macrophage subtypes. Positioned in the “outer layer” of the marginal zone, these macrophages are strategically equipped to efficiently capture blood-borne antigens. These cells express a rich array of selective scavenger and pattern recognition receptors, including scavenger receptor A, CD204, Marco (a macrophage receptor with a collagen structure), and CD209b (SIGN-R1, a DC-SIGN homolog) [29, 100–106]. Research indicates that macrophages within the marginal zone play a pivotal role in regulating the retention of B cells within this region [107]. Additionally, the structural integrity of the splenic marginal zone relies on the presence and activity of B cells [101, 102].

### Fundamental functions of perivascular macrophages

**Fig. 4:**
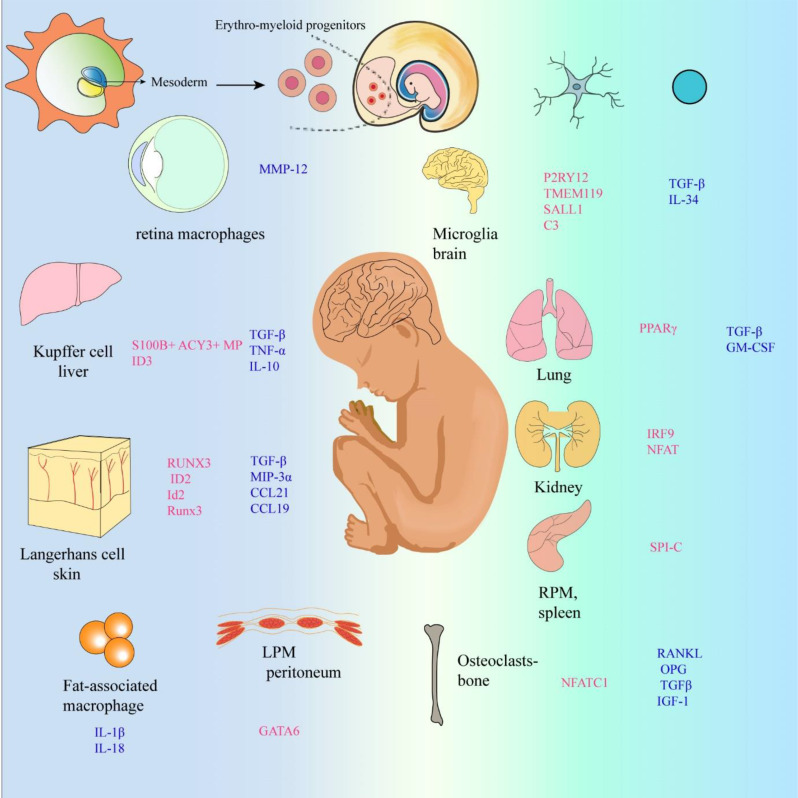
Macrophage ontogeny and specification. Arrows indicate developmental relationships between cells. Macrophage-specific transcription factors and tissue signals are marked in red and blue, respectively. Tissue-resident macrophages are tissue-specific subpopulations that arise during organogenesis. They establish and maintain stable spatial and functional relationships with specialized tissue cells. For example, microglia coexist with neurons in the brain, osteoclasts with osteoblasts in bone, and adipose-associated macrophages with white adipocytes in adipose tissue. They sense and integrate local and systemic information to provide growth factors, nutrient recycling and waste removal to specialized tissue cells essential for tissue growth, homeostasis and repair

Perivascular macrophages (PVMs) are a unique population of macrophages in the brain, mainly distributed in the perivascular space of small arteries and veins with a diameter of 10–35 μm. The shape of PVMs is elongated [108]. Regarding the origin of PVM, according to the recent study by Masuda et al. [109] PVMs are generated in an integrin-dependent manner after birth by migration of chondrocytic macrophages into the perivascular space. CD206 and lymphatic vessel endothelial hyaluronan receptor 1 (Lyve1) are PVM-specific markers. PVMs are relatively stable under physiological conditions and do not normally exchange with peripheral monocytes. However, they can be replenished by peripheral cells either in pathological states or through intervention. In adult homeostatic tissues, PVMs usually have important functions related to their perivascular location, such as regulating vascular permeability, clearing blood-borne pathogens, and controlling the movement of other leukocytes in the vascular system. Although PVMs may express some unique phenotypic markers in some tissues, they do not share a single, common phenotype. This may be due to the exposure of PVMs to different signals in various tissues and possible differences in their individual occurrence [59, 110](Fig. 4).

## The main changes in innate immune cells in neurodegenerative diseases

### The progression of macrophages in Alzheimer’s disease

Alzheimer’s disease (AD) is the predominant cause of dementia, encompassing the majority of dementia cases worldwide. It represents a significant and escalating global health concern, with far-reaching implications for individuals and the society at large. Characteristic hallmarks of AD pathology include the aberrant accumulation of Aβ, the formation of neurofibrillary tangles, neuronal degeneration, and the occurrence of neuroinflammatory processes [111, 112]. Currently, the amyloid cascade hypothesis is the prevailing theory regarding the pathogenesis of AD. According to this hypothesis, dysfunction in the clearance of Aβ is considered the primary factor leading to the accumulation of Aβ in sporadic cases of AD, which represent the vast majority (approximately 99%) of all AD cases [113]. Hence, emphasis on the targeted clearance of Aβ as a crucial therapeutic approach for treating ADis growing [114]. Yu et al. [115] indicated that approximately 40–60% of Aβ originating from the brain is transported to the peripheral nervous system for clearance [113, 116]; rather than focusing solely on controlling the overproduction of Aβ, boosting Aβ clearance within the brain could be a more promising treatment strategy for Alzheimer’s disease [113, 117, 118].

Microglia in pathological states (e.g. AD) are activated by various factors and surface related receptors, such as TREM2 and TLRs. Among them, an increase in the expression level of TREM2 protein promotes the polarization of microglia towards an inflammatory state and enhances the activation of NLRP3 inflammasomes, resulting in an exacerbation of the inflammatory response. TREM2 and TLRs can bind to A β and ApoE and then migrate specifically to the site of injury [43, 119, 120]. In addition, A β aggregates can activate microglia to M1 type through TLRs and RAGE receptors, causing microglia migration and promoting acute and chronic inflammatory responses to aggregates. These receptors, in turn, can activate NF kB and AP-1 transcription factors, inducing the production of NO, ROS, pro-inflammatory cytokines TNF α, IL-1 β, and IL-6, which may ultimately promote neuronal death [43].

There is a growing evidence that perivascular macrophages (PVMs), the other major innate immune cell, are intimately involved in neurodegenerative diseases and act in a protective and/or destructive manner during disease progression. In the context of AD pathology, PVMs are essential for Aβ amyloid phagocytic clearance and is regulated by scavenger receptor class B type I (SR-BI). When PVMs are depleted in TgCRND8 mice using chloroform, Aβ deposition around cerebral blood vessels is increased [121, 122]. Moreover, the absence of SR-BI increase Aβ deposition in the hippocampal region of the hippocampus and in the brain parenchyma of J20 mice, while exacerbating the behavioral deficits in J20 model mice [123]. However, studies have also elaborated on the deleterious role of PVMs in AD. Park et al. [108] found that intravascular Aβ crosses the vessel wall, enters the perivascular space and reaches the PVMs, inducing ROS production and altering neurovascular function. Therefore, the researchers concluded that PVMs are responsible for vascular dysfunction in AD. In addition to its Aβ-related functions, PVM is thought to play a role in blood-brain barrier regulation and antigen.

#### Inflammatory factors related to the AD central nervous system

A study documented notable increases in the levels of proinflammatory cytokines, including TNF-α, IL-6, IL-12p40, IL-1β, IL-1α, and GM-CSF, within the brains of transgenic mice [111]. Recent investigations have identified mutations in AD risk genes associated with the immune response and endocytosis, such as ATP-binding cassette transporter A7, CD33, triggering receptor expressed on myeloid cells 2 (TREM2), and complement receptor 1, suggesting that innate immune dysfunction primarily involves microglia and peripheral myeloid cells [124]. Elevated CD33 expression has been correlated with more pronounced cognitive decline and AD progression.

#### Neuroinflammatory factors secreted by AD-related barrier cells in the immune response

In AD, perturbations in the blood‒brain barrier manifest through the accumulation of circulating molecules, notably fibrinogen, albumin, and IgG. Concurrently, a decrease in the expression of endothelial cell-specific connexins, including claudin-5, occludin, ZO-1, and ve-calmodulin, is observed [125–129]. Moreover, endothelial cell activation, characterized by the upregulation of cell adhesion molecules such as VCAM-1, ICAM-1, E-selectin, and P-selectin, contributes significantly to blood‒brain barrier compromise [129, 130]. Pericytes, integral constituents of the blood vessel wall encircling capillaries, play a pivotal role in maintaining cerebrovascular homeostasis and preserving the integrity of the blood‒brain barrier [131]. Remarkably, cerebral capillaries exhibit a greater density of pericytes than their peripheral counterparts, highlighting their paramount importance in the brain microvascular architecture [131]. Studies utilizing transgenic animal models have underscored the effects of pericyte depletion on exacerbating AD pathology, a phenomenon corroborated by observations of diminished pericyte coverage in postmortem analyses of AD-afflicted individuals [132–134]. Disruption of the blood‒brain barrier, coupled with subsequent protein extravasation, serves as a trigger for microglial activation, initiating inflammatory cascades within the cerebral milieu [129, 135]. Additionally, microglia have been implicated in contributing to vascular injury through the release of proinflammatory cytokines or matrix metalloproteinases (MMPs) into the systemic circulation [129, 136]. However, the precise mechanisms governing microglial modulation of vascular injury, as well as the temporal sequence of events linking microglial activation to blood–brain barrier dysfunction and cerebrovascular impairment, remain subjects of ongoing investigations and scrutiny in the field.

#### AD-related inflammatory factors in the peripheral nervous system

Physiological clearance mechanisms responsible for removing Aβ from the periphery are indispensable for preventing its accumulation in the brain. Multiple organs, tissues, and immune cell subsets contribute to Aβ degradation and catabolism, collectively forming crucial Aβ clearance pathways [118]. Notably, investigations employing human BBB models and AD mouse models have revealed that more than half of the Aβ derived from the brain is naturally eliminated through exocytosis to the periphery [137]. Consequently, a substantial reduction in peripheral Aβ levels or impediments to brain influx results in a net efflux, thereby mitigating Aβ deposition and ameliorating AD-related neuropathological changes in the brain [138, 139]. For instance, studies have shown that peritoneal dialysis effectively reduces plasma Aβ concentrations in both human cohorts and animal models, subsequently alleviating AD-associated phenotypes in mice harboring APP/PS1 mutations [140, 141]. Conversely, renal or hepatic impairment in patients or experimental models correlates with elevated Aβ levels, whereas interventions such as renal dialysis or enhanced hepatic Aβ degradation effectively lower Aβ concentrations [118, 140, 142]. Notably, investigations by Yu et al. [115] and Sevigny et al. [143] have modeled AD and splenectomy, revealing that splenic monocytes/macrophages play a direct role in Aβ uptake both in vivo and in vitro. Furthermore, splenectomy alters the composition of blood immune cell populations, diminishing circulating Aβ uptake and potentially exacerbating Aβ accumulation in the brain. Moreover, splenectomy leads to a reduction in monocyte/macrophage numbers, resulting in elevated blood Aβ levels and suggesting that the spleen is involved in the physiological removal of Aβ from the circulation [144]. Recent findings indicating that senescent splenocytes accelerate aging, a significant risk factor for AD, underscore the potential contributions of peripheral organs and immune cell subsets to Aβ clearance. Genome-wide meta-analyses have highlighted the robust expression of AD risk-associated genes in immune-related tissues and cell types, including the spleen, liver, and microglia, implicating their roles in AD pathogenesis [145]. Consequently, as individuals age or develop AD, their capacity to clear circulating Aβ diminishes significantly. Thus, specific peripheral organs, tissues, and immune cell subsets likely play pivotal roles in Aβ degradation and catabolism, representing promising avenues for targeted Aβ clearance strategies, with monocytes/macrophages emerging as playing key roles in this process [118].

Previous investigations have predominantly focused on the migration of peripheral monocytes/macrophages into the brain or cerebrovascular region to clear central amyloid-beta (Aβ), often overlooking the potential for direct Aβ phagocytosis in the periphery. However, macrophages residing in peripheral tissues and organs, along with circulating monocytes, exhibit robust abilities to engulf Aβ [141]. Given the abundant presence of peripheral monocytes/macrophages, they may serve as an efficient reservoir for peripheral Aβ, effectively sequestering Aβ away from the brain and ameliorating AD pathology [141]. Therefore, augmenting the phagocytic activity of peripheral blood monocytes/macrophages has emerged as a promising strategy to enhance Aβ clearance within the brain.

Xu et al. [141] showed that in AD, Aβ triggers inflammatory activation in macrophages, resulting in the upregulation of proinflammatory markers such as interleukin 1β (IL-1β), interleukin 6 (IL-6), and iNOS. They further elucidated the pivotal role of Smad signaling in macrophage phagocytosis, observing that Smad3 activation levels exhibited a negative correlation with age-related decreases in macrophage phagocytic activity and a positive correlation with AD neuropathological progression. Additionally, Smad3 signaling was found to modulate the inflammatory response, as Smad3 deficiency mitigated NF-κB signaling-dependent inflammation in diabetic nephropathy [141]. Notably, enhancing Aβ clearance by peripheral macrophages through Smad3 inhibition was reported to ameliorate AD-associated Aβ deposition, neuroinflammation, and cognitive deficits, revealing novel insights into AD pathogenesis and potential therapeutic interventions.

### Progress in determining the role of macrophages in PD

Parkinson’s disease (PD) is a prevalent neurodegenerative disorder affecting middle-aged and elderly individuals. In China, the prevalence of PD among those aged over 65 years is estimated to range from 1.6 to 1.7%. Projections indicate that by the year 2030, the number of PD patients in China is expected to exceed 5 million, constituting approximately half of the global PD patient population [146]. PD is characterized by a multifaceted pathophysiology involving various interconnected mechanisms. These mechanisms include the aggregation of α-synuclein, mitochondrial dysfunction, impairment of lysosomal or vesicular transport, synaptic dysfunction, and neuroinflammation. Collectively, these pathological processes culminate in the accelerated death of predominantly dopaminergic neurons. However, the neuropathology of Parkinson’s disease extends beyond dopaminergic circuits, affecting multiple other motor and nonmotor neuronal circuits as well [146]. Alpha-synuclein protofibrils have been shown to elicit microglial activation and trigger inflammatory responses. Additionally, they facilitate phagocytosis, enabling the removal of neurons containing pathological alpha-synuclein aggregates through intercellular membrane protrusions [147]. Alpha-synuclein (α-syn) acts as a damage-associated molecular pattern (DAMP), triggering the activation of myeloid phagocytes, which include microglia, monocyte-derived macrophages, and dendritic cells. This activation process can also be induced by DAMP-associated oxidative stress mediators released by damaged or deceased neurons. These mediators are detected by pattern-recognition receptors (PRRs), such as Toll-like receptor 2 (TLR2), Toll-like receptor 4 (TLR4), and CD11b. The recognition of damage-related signals by PRRs plays a pivotal role in the initiation of oxidative stress and subsequent inflammatory responses [148]. Impaired clearance of extracellular α-synuclein occurs when microglial autophagy is compromised. Moreover, dysfunction of the microglial autophagy‒lysosome system and an imbalance in sphingolipid metabolism can exacerbate the aggregation and seeding of pathological α-synuclein by enhancing the activation of inflammatory vesicles [149]. While microglia are recognized as the primary immune cells in the CNS responsible for responding to neurological injury, studies have revealed the infiltration of peripheral immune cells into the brain of Parkinson’s disease (PD) patients [150]. Grozdanov et al. [151] reported an increase in the number of proinflammatory monocytes characterized by activation of the CCR2-CCL2 axis in individuals with Parkinson’s disease (PD). Additionally, natural killer (NK) cells have been shown to clear α-synuclein aggregates through both the endosomal–lysosomal pathway and the activated antigen-presenting cell pathway [152]. In the context of adaptive immunity, regulatory T cells (Tregs) play a crucial role in mitigating neurotoxicity by inhibiting the release of ROS from neurons and microglia, thus promoting neuroprotection. Conversely, helper T (Th)1 and Th17 cells, through the secretion of interferon (IFN)-γ and interleukin IL-17, respectively, can expedite neuronal death. Notably, the accumulation of pathological α-synuclein has been linked to the induction of Th17 cell differentiation, which impedes the function of Tregs, consequently amplifying the proinflammatory cascade within the nervous system [153]. Pathological α-synuclein triggers activated astrocytes to upregulate the production and release of vascular endothelial growth factor A (VEGF-A) and nitric oxide (NO). This collaborative effect contributes to the disruption of the blood‒brain barrier, a crucial event in the pathogenesis of Parkinson’s disease [154]. Endothelial cells play a multifaceted role in Parkinson’s disease pathophysiology. They can secrete chemokines that promote microglial migration and activity. Additionally, activated T cells can degrade the peri-endothelial basement membrane via endothelial glycosidases, enabling them to breach the BBB and infiltrate brain tissue, exerting their functions there. Recent single-cell transcriptome sequencing studies of peripheral blood mononuclear cells from Parkinson’s disease patients have shed light on the involvement of PD-associated peripheral CD4^+^ T cells in BBB impairment. Specifically, these T cells may contribute to BBB dysfunction by migrating to midbrain endothelial cells and stimulating endothelial cell responses to IFN-γ [155].

### Progress in determining the role of macrophages in MS

Rua et al. [93] underscore the significance of pivotal inquiries that could steer the trajectory of neuroimmunological investigations in the foreseeable future. For instance, the intricate interplay among various CNS-resident cell populations governs their functionality [94]. Hence, the transcriptional alterations observed in MMs following infection may impact not only their subsequent reactions to pathogenic invasion but also their capacity to interact with and modulate the functions of other CNS-resident cells, potentially influencing the onset of CNS disorders. Indeed, various environmental factors, such as infections, are believed to play a role in the pathogenesis of multiple sclerosis (MS) and other similar diseases [95]. Microbial metabolites generated by the commensal gut microbiota have been reported to regulate the function of CNS-resident cells. The boundaries of the CNS make them susceptible to peripheral signals, including microbial metabolites absorbed by the host [95, 156–158]. Hence, alterations in the repertoire of microbial sensing molecules detected following viral infection could influence the ability of microglia/macrophages (MMs) to respond to microbial metabolites, thereby modulating the regulation of CNS inflammation via the gut–brain axis in both health and disease. Furthermore, the symbiotic flora may influence MMs and peripheral monocytes to govern the recruitment and polarization of meningeal monocytes [159]. Consequently, ascertaining whether pathogen-induced changes in MM populations contribute to the onset of MS while also proposing a potential role for alterations in the MM phenotype and function in disease pathogenesis are crucial. Valuable insights into the regulation of meningeal inflammation by MMs and its long-term neurological repercussions are provided, illuminating the pathogenesis of neurological disorders and offering novel therapeutic avenues for clinical intervention.

### ALS-related macrophages

ALS is a multifaceted condition characterized by the progressive degeneration of motor neuron function. This degeneration manifests as the selective demise of motor neurons within the CNS and the subsequent denervation of neuromuscular synapses in the peripheral nervous system (PNS). Disease progression culminates in a debilitating loss of motor function, often leading to paralysis and respiratory insufficiency. ALS has a global prevalence of approximately 8 cases per 100,000 individuals, with most patients succumbing to respiratory failure within 2 to 5 years following diagnosis [160, 161]. Respiratory compromise, attributed to the loss of motor neurons innervating the diaphragm and chest wall muscles, is the primary cause of mortality in individuals with ALS [162]. Studies have underscored the prognostic value of assessing forceful lung capacity (FVC) in predicting survival rates among ALS patients. Despite extensive research efforts, no approved or investigational therapies currently offer reliable prognostic indicators for ALS patients.

Immunopathological mechanisms in amyotrophic lateral sclerosis (ALS) include various processes, such as the phagocytosis of apoptotic and nonapoptotic neuronal cells by inflammatory macrophages, cytotoxic effects mediated by granzyme-positive CD8^+^ T cells, disruption of the blood‒brain barrier by Th17 cells, and IL-6 trans-signaling, which has been shown to exert dose-related toxicity in mouse brains. Additionally, dysregulation of microglia and T cells due to decreased levels of TGF-β, diminished neuroprotection, reduced numbers of regulatory T cells, and a deficiency in trophic factors contributes to the pathogenesis of ALS [163].

ALS is associated with the function of microglia and astrocytes near motor neurons. In ALS related research, Trem2 and Tyrobp mediated signal transduction is an early step in microglial cell disease. In the spatiotemporal dynamic pattern of glial cell proliferation in ALS, the gene expression patterns of microglia such as Trem2 and Tyrobp are correlated with the patterns of Lrp1 and Gba. The Trem2 submodule includes many factors involved in complement cascade, such as Fc receptor mediated signal transduction and phagocytosis. However, the Lrp1 submodule includes many sphingolipid signaling transduction factors [164]. In vitro experiments, researchers have found that mutated mSOD1 protein can also activate microglia, possibly through CD14 (a pattern recognition receptor for misfolded proteins), as CD14 shares common receptors TLR-2 and TLR-4. Similar to LPS, mSOD1 can bind to CD14. After using antibodies against TLR2/4 or CD14, or alternatively blocking this pathway by using CD14^-/-^ microglia, it was found not only the decreased production of pro-inflammatory cytokines and free radicals but also the increased release of IGF-1 from mSOD1G93A microglia, thereby weakening neurotoxicity and enhancing the neuroprotective effect of microglia, which indicates that activation of microglia through the CD14 and TLR pathways is one of the neuropathological markers of ALS. Thus, selectively enhancing or blocking the activation mechanism of microglia may be a potential therapeutic approach [165, 166].

The most prevalent mutations associated with sporadic ALS (SALS) occur in genes such as C9orf72, SOD1, FUS, or TARDBP, accounting for approximately 40%, 20%, 1–5%, and 2–5% of familial ALS (FALS) cases, respectively [167]. The etiology of 90–95% of SALS cases remains elusive, with only 5–10% attributed to known genetic mutations. Familial ALS (FALS) is associated with more than 30 different genes [168, 169]. The current understanding implicates multiple genetic epitopes and mechanisms in SALS, including DNA methylation, histone remodeling, aberrant miRNA genesis, and other silencing mechanisms [170]. Alterations in the CNS expression of genes such as C9orf72, MATR, and VEGFA have been observed [171]. Moreover, transcriptional changes in peripheral blood mononuclear cells (PBMCs) involve the genes B2M, ACTG1, DYNLT1, SKIV2L2, C12orf35, TARDBP, and ILKAP [172]. TBK1, identified as an ALS-associated gene, links the autophagy of ubiquitinated proteins with inflammation [172]. Studies have shown that C9orf72 deletion can upregulate Trem2 and Tyrobp, immune markers possibly linked to intrinsic immunity [173]. The SMCR8 protein forms complexes with C9orf72, which exhibits guanine nucleotide exchange factor (GEF) activity against RAB39B (a member of the RAS oncogene family) and RAB8A (a member of the RAS oncogene family), key regulators of cellular processes such as cytoplasmic reorganization, proliferation, migration, intracellular transport, and differentiation through GTPase activation [174, 175]. In addition, Shao et al. [175] showed that the SMCR8-C9orf72 complex is associated with the autophagy initiation-related complex ULK1 (unc-51 like kinase 1)-ATG13 (autophagy related 13)-RB1CC1/FIP200 (RB1-inducible coiled-coil 1). Moreover, Smcr8-deficient macrophages showed increased expression of NOS2, a hallmark of M1 macrophages. At the same time, M2 macrophages showed decreased expression of the marker ARG1 (arginase 1). When the c9orf72 and smcr8 genes were mutated the proinflammatory cytokine IL6 was elevated in peripheral blood, the number of CD68-positive macrophages in the splenic red pulp increased, and the mRNA expression of the inflammatory cytokines Il-1β and Nos2 increased. Deletion of C9orf72 or Smcr8 resulted in the upregulation of MTOR proteins and the MTORC1 signaling pathway. Thus, researchers have not clearly determined whether altering macrophage activation attenuates the inflammatory response in ALS patients. However, relevant studies have shown that if peripheral macrophages are appropriately regulated at appropriate time, they may have an impact on microglia in the central nervous system, prompting them to enter a more “neuroprotective” state and providing overall therapeutic benefits for improving motor function in amyotrophic lateral sclerosis [176].

The absence of a clear-cut M1/M2 phenotype of microglia observed in patients with ALS has been documented previously [177–179]. Therefore, microglia in symptomatic patients with ALS cannot be definitively classified as either proinflammatory (M1) or alternatively activated (M2) microglia, which are typically associated with acute rather than chronic inflammation. The precise triggers initiating the dysregulated inflammatory response in ALS patients remain elusive. One plausible conjecture is that microglia exhibit delayed responsiveness to inflammation compared to that of macrophages, rendering them less efficient at tissue healing and repair and more inclined to sustain chronic inflammation. This microglial phenotype mirrors findings in Alzheimer’s disease models and experimental autoimmune encephalomyelitis, leading to the terminology “disease-associated microglia” (DAM) [180–184]. This phenotype is elicited through the TREM2 signaling pathway in response to chronic stimulation by neuronal fragments and protein aggregates, among other factors. The DAM phenotype is characterized by the downregulation of homeostatic genes such as CX3CR1, P2RY12, and TMEM119, while the upregulation of APOE, CCL3, CLEC7A, CST7, CTSE, GPNMB, ITGAX, LGALS3, LILRB4, and LPL is observed. These genes are associated with microglial activity, phagocytosis, and inflammation [181]. Some reports suggest an association between chronic inflammation in ALS and TGF-β [178, 185, 186]. TGF-β is primarily synthesized by astrocytes and has diverse immune regulatory functions, including promoting anti-inflammatory responses and regulatory T cells (Tregs) and facilitating proinflammatory Th17 T cells and fibrosis. These effects are context dependent and influenced by the local microenvironment and cytokine profile [178, 186]. In mutant hSOD1-activated microglia, TGF-β induces the differentiation of reactive astrocytes, which in turn inhibits the neuroprotective actions of microglia, contributing to the acceleration of disease progression [178].

Graves et al. [163] reported that inflammation in both the spinal cord and cortex of ALS patients involves innate immune responses orchestrated by macrophages and mast cells, as well as adaptive immune responses mediated by T cells. They observed dense infiltration of macrophages in both the white and gray matter of the ALS spinal cord, which appeared morphologically distinct from microglia, suggesting that these cells originated from the bloodstream [170]. Following macrophage infiltration in the ALS spinal cord and cortex, CD40 ligand-positive T cells infiltrate the spinal cord, initiating adaptive immunity by binding to macrophages expressing the CD40 receptor. ALS spinal cord macrophages exhibit elevated expression of cyclooxygenase-2 (COX-2) and inducible nitric oxide synthase (iNOS), while spinal cord blood vessels display tight junction protein disruption and are surrounded by CD68-positive macrophages, further attracting CD3^+^ T cells. Additionally, a study indicated that downregulation of the neuroprotective M2/Treg/Th2-mediated pathway and upregulation of the cytotoxic M1/Th1/Th17 pathway accelerated the progression of proinflammatory disease [115, 187–189].

Mast cell infiltration is also evident in the ALS spinal cord. These cells, which are present in neural tissue, are known to secrete neurotrophic factors and potentially contribute to regenerative processes [163, 190]. Notably, mast cells are localized in skeletal muscle areas adjacent to tendons, and their distribution throughout the muscle is altered following denervation. Furthermore, on the skin and mucosal surfaces of organs such as the lungs, intestines, and bladder, mast cells are positioned near sensory nerves via a narrow synapse-like gap, enabling intercellular communication mediated by substances such as NT-3 and substance P [163, 191]. Additionally, mast cells can initiate the cytokine cascade response by releasing preformed TNF-α, akin to the process in macrophages. Given their expression of COX-2, similar to that of macrophages, further investigations are warranted to explore the potential neuroprotective effects of COX-2 inhibitors on ALS spinal cord neurons.

## Neurodegenerative diseases and macrophage-related therapeutic drugs and targets

### Potential therapeutic drugs and targets for macrophages in AD

The decrease in monocyte-mediated clearance of circulating amyloid-beta (Aβ) observed in aging individuals with AD underscores a potential therapeutic strategy: enhancing Aβ phagocytosis by monocytes/macrophages. In a recent study, the intraperitoneal administration of polysaccharide hormone (PSK) to APP/PS1 mice improved performance on behavioral tests and reduced Aβ deposition, neuroinflammation, neuronal loss, and tau protein hyperphosphorylation. These findings suggest the potential of PSK as an AD preventive agent by augmenting antibody clearance by blood monocytes and mitigating AD-like pathology [192].

Diminished expression of Toll-like receptor 2 (TLR2), a natural innate immune receptor that recognizes and takes up antibodies via a receptor complex with CD14 polysaccharide kinase, has been noted in individuals with AD. Activation of TLR2 restores antibody uptake by monocytes in individuals with AD, and as a novel TLR2 agonist, PSK may promote Aβ uptake by monocytes through TLR2 activation [193, 194].

Moreover, transplantation of splenocytes from young mice has been shown to delay the aging process in senescent mice [144], suggesting that the spleen is a potential therapeutic target for AD. Considering that DAMPs and misfolded proteins drive neuroinflammation by interacting with various pattern recognition receptors (PRRs), such as RAGE, Mac1, and TLRs, targeting these PRRs may offer therapeutic avenues. In a related study, administration of the RAGE receptor inhibitor PF-04494700 to APP transgenic mice resulted in reduced Aβ peptide accumulation in the spleen, decreased expression of IL-6 and macrophage colony-stimulating factor, and significant reductions in inflammatory markers (TNF-α, TGF-β, and IL-1) and amyloid deposition in the central nervous system [195]. At present, there is no consistent or clinically significant impact of PF-04494700 on plasma A β levels, inflammatory biomarkers, or secondary cognitive or functional outcomes. Further evaluation is needed to assess the impact of long-term treatment on the cognition and clinical measures of AD patients [195].

Recent investigations have implicated NLRP3, an inflammasome effector protein, in AD pathogenesis. NLRP3 forms functionally complete inflammasomes by binding to the pyrin structural domain of the apoptosis-associated speck-like protein ASC, recruiting caspase-1 and subsequently cleaving the proinflammatory cytokines IL-1β and IL-18 into their mature forms. Inhibition of NLRP3 with MCC950 in APP/PS1 transgenic mice led to reduced caspase-1 and IL-1β activation and enhanced Aβ clearance [196, 197]. MCC950 was once promoted to the clinical phase II of rheumatoid arthritis, but it was found to cause an increase in serum liver enzyme levels, and therefore the project was shelved [198]. Twenty years after the discovery of MCC950, Genentech restarted this failed project. It was later discovered that key compounds could accumulate in the kidneys due to pH dependent solubility, ultimately causing kidney damage. Therefore, it is very important to address the side effects caused by this drug [199].

Gantenerumab, a monoclonal antibody designed to target aggregated amyloid-beta (Aβ) in the brain, specifically binds to the N-terminal and intermediate structural domains of Aβ peptides with high affinity for aggregated amyloid beta species. The results from a phase III clinical trial revealed a dose-dependent reduction in Aβ plaques in the brain below the threshold for healthy individuals, accompanied by improvements in patients’ mental status and reductions in cognitive decline, particularly in early disease stages [200, 201]. The researchers conducted two Phase 3 trials (Gradate I and II), and the results showed that after 116 weeks of treatment, the experimental group decreased brain A β levels compared to the control group. Nevertheless, the former group did not delay the progression of AD [202].

In addition, it has been shown to improve patients’ mental status and reduce cognitive decline, especially in the early stages of the disease [14, 201]. Xu et al. [141] documented that inhibiting the Smad3 signaling pathway promotes macrophage phagocytosis and Aβ degradation while inducing macrophage polarization toward an anti-inflammatory phenotype in both primary cell culture and animal models. Smad3 blockade effectively lowered Aβ levels in peripheral organs and the circulation, facilitating Aβ efflux from the brain, likely through enhanced clearance by peripheral macrophages. These processes resulted in reduced Aβ deposition and neuroinflammation in the brain, ultimately ameliorating behavioral deficits in APP/PS1 ADmodel mice. However, Smad3 blockade using SIS3 did not significantly impact microglial phagocytosis of Aβ in the brains of APP/PS1 mice. Instead, SIS3 treatment inhibited microglial activation and local neuroinflammation, possibly due to decreased local Aβ accumulation.

### Potential therapeutic drugs and targets for macrophages in PD

Prasinezumab, also known as PRX002, is a monoclonal antibody directed toward the C-terminal region of alpha-synuclein (α-syn) and is currently undergoing phase II clinical trials. Studies have shown that targeting α-syn aggregates with prasinezumab significantly reduces free serum α-syn levels, albeit without affecting free cerebrospinal fluid (CSF) α-syn levels [14, 203, 204]. Preclinical investigations with the precursor to PRX002, mouse monoclonal antibody 9E4, have shown that the antibody-α-syn aggregation complex leads to decreased α-syn concentrations through Fc-γ receptor-mediated internalization on microglial surfaces [204]. The second phase clinical trial of PASADENA showed a certain slowing effect on the progression of motor symptoms in rapidly progressing cases. In addition, in the baseline subgroup receiving monoamine oxidase B inhibitors (MAO-B), Prasinezumab showed a more significant effect in inhibiting symptom deterioration, whose outstanding performance may be related to the speed and degree of aggregation of alpha synuclein, suggesting that intervention with aggregated alpha synuclein may be more effective in rapidly progressing PD subtypes [205].

BIIB054, another monoclonal antibody, targets the N-terminal region of α-syn and is currently undergoing phase II clinical trials. This antibody preferentially binds to alpha-synuclein aggregates and has been deemed safe and tolerable in clinical studies [14, 206, 207]. Relative studies have shown that BIIB054 analyzes the efficacy of anti alpha synuclein monoclonal antibody Cinpanemab in reducing injury and disability in Parkinson’s disease patients compared to placebo in the Phase 2 clinical study SPARK. However, it does not reach the primary and secondary endpoints, and further development is discontinued. Furthermore, the application of sargramostim (GM-CSF) for Parkinson’s disease (PD) treatment has been linked to the gene and/or protein expression levels of potential biomarkers such as LRRK2, HMOX1, TLR2, TLR8, RELA, ATG7, and GABARAPL2, which may predict therapeutic responses to immunomodulatory therapies [148].

Exenatide, a synthetic glucagon-like peptide 1 (GLP-1) agonist, has shown promising results in a randomized, placebo-controlled clinical trial involving patients with moderate PD. After 12 weeks of exenatide exposure, improvements in motor function and disease severity, particularly in exercise severity, were observed after the discontinuation of nocturnal dopaminergic medication [208]. GLP-1 is an enteric insulinotropic hormone secreted mainly by intestinal L cells. GLP-1 has beneficial effects on glucose homeostasis, stimulates B cell proliferation and differentiation, and inhibits B cell apoptosis. Lef-1, a GLP-1 receptor agonist, e.g., liraglutide (50 lg/kg, s.c.), was administered with a subcutaneous injection of fisetinone (3 mg/kg/d, s.c.) to male albino rats for 16 days and was found to have a statistically significant effect on behavioral activity, resulting in a statistically significant increase in striatal levels of dopamine and a neurotrophic factor of nigral glial cell line origin (GDNF); tyrosine hydroxylase positivity (TH^+^); and decreased levels of the proinflammatory factors interleukin (IL)-1β, IL-6, and transforming growth factor (TGF)-β1 [209, 210]. A phase II clinical trial showed that Lixisenatide (lisenatide, developed by Sanofi), a GLP-1 receptor agonist for the treatment of diabetes, could slow down the progress of Parkinson’s disease related dyskinesia after 12 months of treatment [210].

Gut microbial dysbiosis is suggested to alter TLR2 and TLR4 signaling, promoting α-synuclein aggregation in enteric and vagal neurons, which in turn migrates to the brain via peripheral nerves and contributes to neurodegeneration. These results provide a strong evidence for the involvement of gut microbial dysbiosis in PD development [211, 212]. There is an ongoing Phase 1 clinical study being conducted on TB006.

TLR2 activation further enhances the aggregation of α-synuclein by regulating autophagy. In line with these results, blocking the interaction between TLR2 and MyD88 can reduce the activation of glial cells, decrease α-synuclein spreading, and protect dopaminergic neurons [211, 213, 214]. Currently, a phase 1 clinical study is being conducted on healthy volunteers with NPT520-34.

### Potential therapeutic drugs and targets for macrophages in ALS of the spinal cord

ALS is a debilitating neurodegenerative condition characterized by the progressive degeneration of motor neurons, leading to paralysis and long-term disability. Currently, riluzole is the sole FDA-approved medication for ALS treatment [215, 216]. The neuroprotective effects of IGF-1 are mediated by downstream signaling pathways involving insulin receptor substrates 1 and 2 (IRS1 and IRS2), followed by activation of the PI3K/Akt and p44/42 MAPK pathways [217, 218]. Activity-dependent neurotrophic factor (ADNF)-derived peptides have also produced neuroprotective effects on ALS-SOD1 mouse models.

These signaling mechanisms lead to the neuroprotective effects of IGF-1 [219]. Intramuscular injection of an IGF-1-expressing adeno-associated virus (AAV) delays disease progression and prolongs the lifespan of a transgenic mouse model of ALS expressing the G93A SOD1 transgene [220]. The retrograde transport of AAV to the cell body via axons facilitates the targeted delivery of IGF-I, resulting in positive outcomes such as muscle atrophy, reduced astrocyte hyperplasia, delayed motor neuron loss, and increased muscle mass [221, 222]. Thus, the use of neurotrophic factors either as monotherapy or in combination therapies holds promise as a potential therapeutic strategy for ALS treatment.

Talampanel, an oral noncompetitive antagonist of the α-amino-3-hydroxy-5-methyl-4-isoxazolepropionic acid (AMPA) receptor, has been identified as a potential therapeutic agent for ALS [223]. AMPA receptors play a crucial role in mediating glutamate-induced excitotoxicity in motor neurons, a key factor in the neuropathogenesis of ALS [224]. In vivo studies utilizing the SOD1 mouse model have revealed the beneficial effects of AMPA antagonists such as talampanel [225]. However, the neuroprotective effects of talampanel are most pronounced when it is administered early in the course of the disease.

In a phase II clinical trial involving 60 ALS patients, Yacila et al. [215] investigated the efficacy, safety, and tolerability of talampanel. While no significant differences were noted in efficacy measures, a slower decline in the ALS functional rating scale score, muscle strength, and timed hand movements was observed.

The impact of TLR4 on ALS has also been studied. An analysis of post-mortem tissue from sporadic ALS patients showed an increased TLR4 expression in the spinal cord [226]. In the hSOD1 G93A transgenic mouse model, the expression of TLR4 is upregulated in microglia and astrocytes, and TLR4 deficiency or antagonist treatment decreases microglia activation, improves motor function, and extends life expectancy [211, 227, 228]. There is a Phase 1 clinical study currently being conducted on TB006. TLR2 expression in microglia is also enhanced in ALS mouse model. The increased expression of TLR2 is strongly associated with aggravated neuroinflammation and degeneration of motor neurons [229]. Furthermore, TLR2 expression is enhanced in the post-mortem spinal cord tissue from sporadic ALS patients [226]. Currently, research is underway to evaluate the safety, pharmacokinetics, and target binding of NPT520-34 in Phase 1 clinical trials targeting healthy volunteers.

Beta-lactam antibiotics have emerged as another potential treatment option for ALS. Synaptic glutamate reuptake, which is crucial for maintaining glutamate homeostasis, relies on normal levels of major glial glutamate transporter 1 (GLT1) [230]. Reduced levels of GLT1 have been observed in ALS patients, potentially leading to diminished glutamate elimination from neuromuscular synapses [231]. Rothstein et al. [232] reported that ceftriaxone, a beta-lactam antibiotic, exerts neuroprotective effects by stimulating the GLT1 promoter sequence, thereby reducing glutamate excitotoxicity. This effect has been associated with the preservation of muscle stability, body weight, and a moderate extension of lifespan [215] (Table 1).

**Table 1:** Summary of therapeutic medicines and their therapeutic targets in neurodegenerative diseases and macrophage-related therapeutics

Diseases and medicines	Consequences	Inflammatory factor changes	Application models	Pathway / Site of action
AD				
SIS3	Perhaps by increasing the phagocytic activity of macrophages and degradation of A β in peripheral blood.	SIS3 treatment significantly reduces TNF-α and IL-6 expression and significantly upregulates IL-10 expression in the cortex and hippocampus.	APP/PS1	Inhibition of Smad3 induced a phenotypic shift in macrophage polarization toward an anti-inflammatory phenotype and significantly enhanced their phagocytosis of Aβ.
PSK	Enhancement of antibody clearance by blood monocytes and attenuation of AD-like pathology with reduced Ab deposition, neuroinflammation, neuronal loss and tau protein hyperphosphorylation.	Decreased TGF-β1.	APP/PS1	Activates the TLR2 receptor; activates the endosomal–lysosomal pathway; promotes Ab uptake by monocytes.
Inhibitor of RAGE, PF-04494700	Inhibits the binding of sRAGE to Aβ1–42; reduces the accumulation of Aβ peptides in the spleen.	Reduced expression of IL-6 and macrophage colony-stimulating factor. Associated with decreased TNF-α, TGF-β and IL-1 levels.	APP	Inhibits the binding of sRAGE to RAGE ligand, S100b, amphiphiles and carboxymethyl lysine.
NLRP3 inhibitor MCC950	Skews microglia toward the M2 phenotype, with reduced deposition of Aβ.	Enhanced Aβ clearance. Reduced IL-1β and IL-18.	APP/PS1	Inhibits LPS- and Aβ-induced activation of caspase 1 and is involved in promoting the phagocytosis of Aβ.
Gantenerumab	Aβ plaque reduction; ameliorated cognitive decline.	–		Aβ N-terminus; A β Middle domain.
PD				
Prasinezumab (PRX002)	Reduced intracellular accumulation of alpha-nucleoprotein in the cell body and at synapses, preventing synaptic loss and glial cell proliferation, and ameliorating motor and cognitive behavioral deficits.	–	A-syn Tg mice	Acts on the α-syn C-terminus.
BIIB054	Attenuating the spread of alpha-syn pathology rescued dyskinesia and reduced the loss of striatal dopaminergic terminal dopamine transporter density.	–	α-syn PFF inoculation of mouse models	α-syn N-terminus; α-syn polymer
Sargramostim	Increased Treg number and function, improved motor function and associated brain activity.	IL-8, ILK, TNF, nitric oxide and oxidative phosphorylation were decreased and the LRRK2, HMOX1 and TLR2 proteins were significantly downregulated.	–	–
Exenatide	Beneficial effects on motor function.	TNF-α and IL-1 alterations.		IRS-1, altered p-Tyr protein signaling; changes in the Akt and mTOR signaling pathways.
Liraglutide	Improved motor function; attenuated nigrostriatal neuron loss.	Reduces levels of interleukin (IL)-1β, IL-6, transforming growth factor (TGF)-β1.	Male albino rats	Significant increases in striatal dopamine and glial cell line neurotrophic factor (GDNF) originating in the substantia nigra; tyrosine hydroxylase positive (TH^+^).
TB006			TLR4 gene (KO)	TLR4 signaling.
NPT520-34	Reduce the aggregation of α-synuclein; protect dopaminergic neurons.			TLR2 signaling.
ALS				
Sargramostim		Decreased IL-8, ILK, TNF, nitric oxide and oxidative phosphorylation levels.		
ACI-5891	Reduced TDP-43 pathology.		rNLS8 mouse	TDP-43-C-terminus-specific monoclonal antibody.
Neurotrophic factor				
IGF-1	Regulation of survival and differentiation and maintenance of neuronal structural integrity.	Improves muscle atrophy and reduces astrocyte proliferation.	SOD1 transgenic mice	PI3K/Akt and p44/42 MAPK.
GDNF				Akt signaling pathway.
VEGF	Stimulation of neurogenesis with potential anterograde and retrograde transit.			
ADNF	Preserves neuronal function but does not prolong neuronal survival.			
Talampanel	Reduction of glutamate-induced excitotoxicity to motor neurons. Improvement of ALS motor symptoms.		SOD1 transgenic mice	Antagonism of AMPA receptors.
Beta-lactam antibiotics	Reduces glutamate excitotoxicity, maintains muscle stability and body weight; moderately extends lifespan.		SOD1 transgenic mice	Promotes GLT1 promoter expression.
TB006	improved motor function and extended life expectancy.		hSOD1 G93A transgenic mouse	TLR4 signaling.
NPT520-34	Reduced the activation of microglia.		SOD1 transgenic mice	TLR2 signaling.

## Emerging strategies for macrophage therapy in neurodegenerative diseases

The burgeoning comprehension of microglial/macrophage phenotypes has ushered in novel therapeutic avenues for CNS remodeling. Notably, M2 microglia/macrophages are heralded for their pro-regenerative properties, representing a promising avenue for cell-based regenerative strategies. In this regard, the transplantation of ex vivo activated M2 cells has emerged as a potential approach to counteract the transition from the M2 phenotype to the M1 phenotype observed in the later stages of injury. Encouragingly, studies using multiple sclerosis models have shown the efficacy of M2 macrophage transplantation in promoting neurological recovery [70, 233].

However, the sensitivity of microglia/macrophages to their extracellular milieu poses a challenge, as unforeseen environmental cues post cell preparation and transplantation may compromise or attenuate their protective M2 features. The application of genetic engineering techniques to generate M2 microglia/macrophages ex vivo holds promise to circumvent this hurdle. This approach may offer greater control over the phenotype and functionality of transplanted cells, enhancing their therapeutic potential. Nonetheless, further comprehensive animal studies are imperative to ascertain the clinical viability of this cell therapy strategy.

In addition to cell transplantation, the secretion of protective factors by M2 microglia/macrophages presents an alternative avenue to bolster regenerative therapies. By harnessing the reparative properties of these cells, efforts can be directed toward augmenting the production of beneficial factors that facilitate CNS repair and regeneration. This multifaceted approach holds considerable promise for advancing the field of regenerative medicine and addressing the unmet needs of patients with CNS disorders (Table 2).

**Table 2:** Macrophage phenotypes and characteristics

Phenotype	Stimuli	Cytokines, chemokines, and other secreted mediators	Functions	Receptors
M1	IFN-γ, LPS	TNF-α, IL-6, IL-12, CCL2, MMP-2, MMP-9	Participates in endocytosis	IFN-γR, TLR, MHCII, CD86
M2	IL-4, IC, LPS	IL-10, IL-4, IL-5, IL-13	Mediates inflammation resolution, promotes tissue repair or remodeling	CD163, MR, GR, SR, IL-4Rα
M2a	IL-4, IL-13	IL-10, TGF-β, CCL17, CCL18, CCL22	Anti-inflammatory diseases, tissue remodeling	CD206, IL-1RA
M2b	ICs, TLR ligands, IL-1 receptor ligands	IL-10, IL-1β, IL-6, and TNF-α	Th2 activation, immunoregulation	TLR, IL-1R
M2c	Glucocorticoids, IL-10	IL-10, TGF-β, CCL16, and CCL18	Apoptosis, cell phagocytosis	CD14, CD206
M2d	TLR ligands, adenosine receptor ligand	IL-10, VEGF	Angiogenesis, tumor progression	TLR, CD14, CD206

## Conclusions

The field of neuroimmunology is rapidly advancing alongside the progress in microglia and CNS-associated macrophage research. While traditional macrophages have long been recognized for their role in inflammation and pathogen clearance, it is now widely accepted that a key aspect of macrophage biology is the maintenance of tissue homeostasis and organ function. In fact, most tissues have their own resident macrophages dedicated to this purpose. The primary focus lies in determining the factors that dictate the tissue identity of specific organs, as well as understanding the extent to which macrophage function can be influenced, rather than being solely determined by a specific developmental lineage.

Disruptions to homeostasis caused by inflammation, diet, injury, and aging may result in bone marrow-derived monocytes replacing embryonic derived monocytes. Furthermore, there is still much to be studied regarding potential functional differences among seemingly “identical” cell types of different origins. Plasticity and flexibility are fundamental characteristics of mononuclear phagocytes and their activation status. It has been observed that the phenotype of polarized M1-M2 macrophages can be partially reversed both in vitro and in vivo.

Additionally, pathological conditions are often associated with dynamic changes in macrophage activation. Conventionally activated M1 cells are believed to play a role in initiating and perpetuating inflammation, while M2 or M2-like cells are linked with resolving inflammation or managing chronic inflammation. However, it remains unclear whether these transformations involve recruiting circulating precursor cells or re-educating existing cells within the tissue itself.

The acknowledged plasticity and diversity of mononuclear phagocytes underscore their ability to integrate a wide array of signals, including those from microbes, injured or dying cells, and the tissue microenvironment. Three distinct pathways control macrophage polarization: epigenetic and cell survival mechanisms, signals from the tissue microenvironment, and external factors such as microbial products and inflammatory cytokines. The significance of macrophage polarization in both normal physiology and pathophysiology has garnered increased attention in recent years. However, questions persist regarding the origins of macrophage heterogeneity, their microanatomical specialization, and how different macrophage populations modulate their epigenetic and phenotypic profiles.

With the aging of the population, the incidence rate of neurological related diseases in China continues to increase. The detection of biomarkers related to neuroinflammation, neurodegenerative diseases, and rare diseases has become one of the important detection methods in the field of neuroscience. There are currently multiple methods available for detecting cytokines, which can be roughly divided into four categories based on their principles and methods: immunological methods, biological methods, molecular biology methods, and mass spectrometry. Among these approaches, immunological methods include Western Blot, enzyme-linked immunosorbent assay (ELISA), ELISA spot technology, hypersensitive electrochemiluminescence technology, Luminex liquid phase chip detection technology Olink technology, and Simoa technology. ELISA is commonly used in clinical practice for qualitative analysis. Mass spectrometry is mainly applied in protein analysis in scientific laboratories such as biomarker discovery and structural identification with a focus on protein expression level analysis.

With the widespread application of new technologies such as single-cell RNA sequencing, errors have been reduced without relying on cell surface markers. Meanwhile, such new technologies may reveal new heterogeneity features in tissue macrophage populations. Nevertheless, there are still many unsolved mysteries regarding macrophage polarization and its role in health and patients.

While studies suggest that embryonic and adult precursors produce transcriptionally similar macrophages, aging, illness, and disease may alter this paradigm. Understanding how brain homeostasis influences macrophage behavior, particularly in the context of inflammation and neurodegenerative diseases, is crucial.

Moreover, the multifaceted functions of brain macrophages, beyond their phagocytic roles, remain largely unexplored. The interactions between microglia/macrophages and other immune cells, such as T and B cells, in health and disease warrant further investigation.

Overall, macrophage plasticity plays a paradoxical role in neurodegenerative diseases, with the potential to either promote or inhibit disease progression. A deeper understanding of the pharmacological and pathological mechanisms underlying macrophage regulation of disease development could pave the way for novel therapeutic interventions for neurodegenerative diseases.

## Data Availability

No datasets were generated or analysed during the current study.

## Abbreviations

Aβ: Amyloid-β
AAV: Adeno-associated virus
AD: Alzheimer’s disease crophages
BAT: Brown adipose tissue
BBB: Blood–brain barrier
CLEC7A: C-type lectin structural domain family 7 member A
CNCCs: Cranial neural crest cells
CNS: Central nervous system
CRP: C-reactive protein
CR3: Complement receptor 3
CSF1R: Colony-stimulating factor 1 receptor
CX3CR1: CX3C chemokine receptor 1
DAMPs: Damage-associated molecular patterns
DCs: Dendritic cells
DD: Death domain
EMPs: Erythro-myeloid progenitors
GEF: Guanine nucleotide exchange factor
GLP-1: Glucagon-like peptide 1
GLT1: Glutamate transporter 1
GM-CSF: Granulocyte-macrophage colony-stimulating factor
HeMPs: Head-enriched Mf progenitors
HSCs: Hematopoietic stem cells
IA: Immunoassays
ICs: Immune complexes
IFN-γ: Interferon-gamma
IGF1: Insulin-like growth factor 1
IL-1: Interleukin-1
IL-1ra: IL-1 receptor antagonist
iNOS: Inducible nitric oxide synthase
IRAK: Interleukin-1 receptor-associated kinase
IRF8: Interferon regulatory factor 8
ITIMs: Immunoreceptor tyrosine-based inhibitory motifs
KO: Knockout
LCMV: Lymphocytic choroid plexus meningitis virus
LCs: Langerhans cells
LFA-1: Lymphocyte function-associated antigen-1
LPMs: Leptomeningeal macrophages
LPS: Lipopolysaccharides
Lyve1: Lymphatic vessel endothelial hyaluronan receptor 1
MCU: Mitochondrial calcium unidirectional
Mf: Macrophage
MMPs: Matrix metalloproteinases
MMs: Meningeal macrophages
MR: Mannose receptor
MRI: Magnetic resonance imaging
mTOR: Mammalian target of rapamycin
MyD88: Myeloid differentiation factor 88
NCCs: Neural crest cells
NF-κB: Nuclear factor kappa-light-chain-enhancer of activated B cells
NK: Natural killer
OX-LDL: Oxidized low-density lipoprotein
PAMPs: Pathogen-associated molecular patterns
PBMCs: Peripheral blood mononuclear cells
PCW: Postconceptional weeks
PD: Parkinson’s disease
PerC: Peritoneal cavity
PGE: Prostaglandin E
PNS: Peripheral nervous system
PPAR-γ: Peroxisome proliferator-activated receptor gamma
PraMs: Primitive macrophages
PreMacs: Macrophage precursors
PRRs: Pattern recognition receptors
PSK: Polysaccharide hormone
PVMs: Perivascular macrophages
RAGE: Receptor for advanced glycation end products
RAR: Retinoic acid receptor
ROS: Reactive oxygen species
SASP: Senescent associated secretory phenotype
scRNA-seq: Single-cell RNA sequencing
SPIO: Superparamagnetic iron oxide
SPMs: Small peritoneal macrophages
SR-A1: Scavenger receptor A1
SR-BI: Scavenger receptor class B type I
SRs: Scavenger receptors
TAK1: β-Transforming growth factor-activated protein kinase
TGFβ: Transforming growth factor beta
TGF-βR: Transforming growth factor-β receptor
TIR: Toll/interleukin-1 receptor
TLRs: Toll-like receptors
TNF-α: Tumor necrosis factor-alpha
TPM: Two-photon excitation fluorescence microscopy
TRAF6: Tumor necrosis factor receptor-associated factor 6
UCP1: Uncoupling protein 1
VEGF: Vascular endothelial growth factor
WAT: White adipose tissue
YSdMPs: Yolk sac-derived macrophage precursors
α-syn: Alpha-synuclein
